# Immunoglobulin G1 Fc glycosylation as an early hallmark of severe COVID-19

**DOI:** 10.1016/j.ebiom.2022.103957

**Published:** 2022-03-22

**Authors:** Tamas Pongracz, Jan Nouta, Wenjun Wang, Krista E. van Meijgaarden, Federica Linty, Gestur Vidarsson, Simone A. Joosten, Tom H.M. Ottenhoff, Cornelis H. Hokke, Jutte J.C. de Vries, Sesmu M. Arbous, Anna H.E. Roukens, Manfred Wuhrer, BEAT-COVID, COVID-19 groups

**Affiliations:** a Center for Proteomics and Metabolomics, Leiden University Medical Center, Leiden, Netherlands; b Department of Experimental Immunohematology, Sanquin Research, Amsterdam, Netherlands; c Landsteiner Laboratory, Amsterdam University Medical Center, Amsterdam, Netherlands; d Department of Infectious Diseases, Leiden University Medical Center, Leiden, Netherlands; e Department of Parasitology, Leiden University Medical Center, Leiden, Netherlands; f Department of Medical Microbiology, Leiden University Medical Center, Leiden, Netherlands; g Department of Intensive Care, Leiden University Medical Center, Leiden, Netherlands

**Keywords:** IgG glycosylation, Anti-spike IgG, SARS-CoV-2, COVID-19, Coronavirus

## Abstract

**Background:**

Immunoglobulin G1 (IgG1) effector functions are impacted by the structure of fragment crystallizable (Fc) tail-linked *N*-glycans. Low fucosylation levels on severe acute respiratory syndrome coronavirus 2 (SARS-CoV-2) spike (S) protein-specific IgG1 has been described as a hallmark of severe coronavirus disease 2019 (COVID-19) and may lead to activation of macrophages via immune complexes thereby promoting inflammatory responses, altogether suggesting involvement of IgG1 Fc glycosylation modulated immune mechanisms in COVID-19.

**Methods:**

In this prospective, observational single center cohort study, IgG1 Fc glycosylation was analyzed by liquid chromatography-mass spectrometry following affinity capturing from serial plasma samples of 159 SARS-CoV-2 infected hospitalized patients.

**Findings:**

At baseline close to disease onset, anti-S IgG1 glycosylation was highly skewed when compared to total plasma IgG1. A rapid, general reduction in glycosylation skewing was observed during the disease course. Low anti-S IgG1 galactosylation and sialylation as well as high bisection were early hallmarks of disease severity, whilst high galactosylation and sialylation and low bisection were found in patients with low disease severity. In line with these observations, anti-S IgG1 glycosylation correlated with various inflammatory markers.

**Interpretation:**

Association of low galactosylation, sialylation as well as high bisection with disease severity and inflammatory markers suggests that further studies are needed to understand how anti-S IgG1 glycosylation may contribute to disease mechanism and to evaluate its biomarker potential.

**Funding:**

This project received funding from the European Commission's Horizon2020 research and innovation program for H2020-MSCA-ITN IMforFUTURE, under grant agreement number 721815, and supported by Crowdfunding Wake Up To Corona, organized by the Leiden University Fund.

**Research in context**

**Evidence before this study**

Antibody glycosylation against the spike (S) protein of patients infected with severe acute respiratory syndrome coronavirus 2 (SARS-CoV-2) has been reported as a potentially important determinant of coronavirus disease 2019 (COVID-19) disease severity. Studies have hitherto focused on afucosylation, a modification on immunoglobulin G1 (IgG1) Fc-tail-linked *N*-glycans that enhances effector functions. Most of these studies featured limited sample numbers or were imperfectly matched with respect to demographic and other important confounding factors. Our lab has contributed to some of these studies, and we additionally searched for research articles on PubMed and Google Scholar from January 2020 to October 2021. To date, only two groups studied anti-S IgG1 glycosylation, which resulted in overall three publications found. However, none of these groups found a severity marker between hospitalized non-ICU and ICU patients or studied dynamic changes. Instead, exclusively fucosylation at the first available timepoint has been associated with disease severity between severely ill inpatients and mild outpatients.

**Added value of this study**

In this prospective, observational single center cohort study, we investigated the severity marker potential of anti-S IgG1 glycosylation in severe and mild hospitalized COVID-19 cases, and correlated these findings with numerous inflammatiory and clinical markers. Our study reveals low galactosylation and sialylation as well as high bisection on anti-S IgG1 as early hallmarks of severe COVID-19, after correction for known confounders of glycosylation. In line with these observations, anti-S IgG1 glycosylation correlated with many inflammatory markers. As days since onset is one of the major confounders of anti-S IgG1 glycosylation due to its highly dynamic nature, we additionally confirmed our findings in time-matched patient subgroups. We believe anti-S IgG1 glycosylation, in combination with other inflammatory markers conveys early severity marker potential in hospitalized patients in this study.

**Implications of all available evidence**

Demographic factors as well as temporal differences should be taken into consideration when analyzing IgG1 glycosylation of COVID-19 patients. Anti-S IgG1 glycosylation is highly dynamic, but is a promising early severity marker in COVID-19.

Alt-text: Unlabelled box

## Introduction

The current global coronavirus disease 19 (COVID-19) pandemic caused by the novel coronavirus severe acute respiratory syndrome coronavirus 2 (SARS-CoV-2) has been leading to extensive hospitalizations worldwide.^1^ To date, more than 253 million infections and more than 5 million deaths have been reported.^2^ SARS-CoV-2 is an enveloped virus and its uptake by target cells in the respiratory tract is mediated by the spike glycoprotein.^1^ Interestingly, most infected people clear the virus with mild symptoms, whilst around 20% of the adult cases are characterized by severe, sometimes life-threatening conditions.^3^ Approximately 7-10 days after symptom onset, seroconversion occurs with immunoglobulin M (IgM) and A (IgA), and G (IgG) antibodies against the spike protein.^4^ These antibodies can form immune complexes with viral particles and thereby neutralize the virus and mediate clearance, but are also capable of aggravating the disease.^5^, ^6^, ^7^

IgG exerts effector functions via the activation of complement or fragment crystallizable (Fc) gamma receptors (FcγR) on immune cells.^8^ Various effector functions of IgG are steered by the *N*-glycan moiety attached to the highly conserved N297 glycosylation sites within both C_H_2 domains of the Fc tail.^9^^,^^10^ Specifically, afucosylated IgG1 shows increased affinity to the activating FcγRIIIa receptor, hence leading to enhanced antibody-dependent cellular cytotoxicity (ADCC).^10^^,^^11^ Galactosylated IgG1 shows increased hexamerization, C1q binding and complement activation.^12^

Recent reports have indicated that the high inter-individual variability in COVID-19 disease severity^3^ may partly be explained by low Fc fucosylation of anti-SARS-CoV-2 spike protein-specific (anti-S) IgG1. The lack of core fucose on these specific antibodies early on during disease points to their potential proinflammatory role in severe illness.^6^^,^^13^^,^^14^ Literature suggests, that in particular membrane-embedded foreign antigens, such as the SARS-CoV-2 spike protein, induce low fucosylated IgG1 responses, which in combination with high titers may lead to excessive macrophage activation and drive COVID-19 associated pathology including acute respiratory distress syndrome.^6^^,^^13^

Here, we study the dynamics of IgG1 Fc glycosylation and its association with clinical parameters in a longitudinal cohort of 159 hospitalized COVID-19 patients that were either admitted to the intensive care unit (ICU) or not (non-ICU), analyzing a total of 1300 longitudinal patient samples. We report on the association of early anti-S IgG1 glycosylation signatures with disease severity and various inflammatory markers.

## Methods

### Chemicals, reagents and enzymes

Type I Ultrapure Water was produced by an ELGA Purelab Ultra system (Elga LabWater, High Wycombe, United Kingdom) and used to create solutions throughout. Ammonium bicarbonate, potassium chloride, formic acid, tolylsulfonyl phenylalanyl chloromethyl ketone-treated trypsin from bovine pancreas was obtained from Sigma-Aldrich (Steinheim, Germany). Trifluoroacetic acid, disodium hydrogen phosphate dihydrate, potassium dihydrogen phosphate, and sodium chloride were purchased from Merck (Darmstadt, Germany). HPLC-supra-gradient acetonitrile was obtained from Biosolve (Valkenswaard, The Netherlands). The Visucon-F pooled healthy human plasma standard originated from Affinity Biologicals (Ancaster, Canada). Protein G Sepharose 4 Fast Flow beads were obtained from GE Healthcare (Uppsala, Sweden). Recombinant trimerized S protein was prepared as described.^15^

### Study cohort

BEAT-COVID-19 is a prospective, observational single center cohort study established at Leiden University Medical Center, with longitudinal plasma samples of 159 polymerase chain reaction (PCR)-confirmed SARS-CoV-2 infected hospitalized patients that were collected during the first and second wave of the pandemic (between May 2020 and October 2020) **(**Tables 1 **and S1, Figure S1)**. After informed consent was obtained from the patient or his/her relatives, longitudinal sampling was performed for the duration of the hospital admission, and one convalescent sample was obtained at the outpatient follow-up appointment, which was scheduled six weeks after hospital discharge. None of the patients had received a COVID-19 vaccine, nor had they used hydroxychloroquine. Patients were hospitalized when their peripheral oxygen saturation was below 92% and they were consequently in need of extra oxygen supplementation via nasal cannula or non-rebreather mask. Patients were discharged when they were not in need of oxygen supplementation anymore and capable of taking care of themselves, irrespective of a potential PCR result. The convalescent sample was 6 weeks after discharge, but not necessarily 6 weeks after a negative PCR. Statistical sample size calculation was not performed, the sample size was determined based on availability. The Medical Ethics Committee Leiden-Den Haag-Delft (NL73740.058.20) approved the study. The trial was registered in the Dutch Trial Registry (NL8589). The study complied with the latest version of the Declaration of Helsinki.

### Sample preparation for IgG Fc glycosylation analysis

Anti-S IgG was captured using a setup that resembles a conventional ELISA: IgGs were affinity-captured from plasma using recombinant trimerized S protein-coated Maxisorp NUNC-Immuno plate (Thermo Fisher Scientific, Roskilde, Denmark), whereas total IgG was affinity-captured using protein G Sepharose Fast Flow 4 beads, as described previously.^13^^,^^16^ Antibodies were eluted using 100 mM formic acid and the samples were dried by vacuum centrifugation. Samples were reconstituted in 25 mM ammonium bicarbonate and subjected to tryptic cleavage, as described elsewhere.^16^ Samples belonging to a single patient were prepared and measured consecutively on the same plate, except for follow-up samples after hospitalization period. On each plate, at least 3 Visucon-F plasma standards (dating pre-COVID-19) and 3 blanks were included.

### IgG Fc glycosylation analysis

Glycopeptides were separated and detected using an Ultimate 3000 high-performance liquid chromatography (HPLC) system (Thermo Fisher Scientific, Waltham, MA) hyphenated to an Impact quadrupole time-of-flight mass spectrometer (Bruker Daltonics, Billerica, MA), as described.^16^ Using this method, IgG1 glycoforms were assigned based on accurate mass and specific migration position in liquid chromatography, excluding the possible glycopeptide-level interference of IgG3 with IgG2 and IgG4.^16^

### Liquid chromatography-mass spectrometry data processing

MzXML files were generated from raw liquid chromatograph – mass spectrometry (LC-MS) spectra. An in-house developed software, LaCyTools was used for the alignment and targeted extraction of raw data.^17^ Alignment was performed based on the average retention time of minimum three abundant IgG1 glycoforms. The targeted extraction list included analytes of the 2^+^ and 3^+^ charge states and was based on manual annotation of the mass spectra as well as on literature.^18^^,^^19^ A pre-COVID-19 plasma pool (Visucon-F) was measured in triplicate in each plate to assess method robustness and was as well used as negative control. All spectra below the average intensity plus three times the standard deviation of negative controls was excluded from further analysis. Signals were integrated by covering a minimum of 95% of the area of the isotopic envelope of glycopeptide peaks. Inclusion of an analyte for the final data analysis was based on quality criteria such as signal-to-noise (> 9), isotopic pattern quality (< 25% deviation from the theoretical isotopic pattern), and mass error (within a ± 20 ppm range). Furthermore, analytes that were present in at least 1 out of 4 anti-S IgG1 spectra (25%) were included in the final analysis.

### Cytokine measurements by cytometric bead array

Circulating cytokine and chemokine levels were determined in serum using commercially available bead-based multiplex assays using the BioPLex 100 system for acquisition as previously described.^20^ Standard curves were included in the kits and, in addition, a pooled serum sample of 4 hospital admitted COVID-19 patients was included as internal reference in all assays. Four commercially available kits were used: Bio-Plex Pro™ Human Cytokine Screening Panel 48-plex, Bio-Plex Pro^tm^ Human Chemokine Panel 40-Plex, Bio-Plex Pro^tm^ Human Inflammation Panel 1 and 37-Plex; Bio-Plex Pro^tm^ Human Th17 panel (all obtained from Bio-Rad, Veenendaal, The Netherlands).

### Antibody titer measurement

Semi-quantitative detection of SARS-CoV-2 anti-nucleocapsid (N) protein IgG was performed on the Abbott Architect platform.^21^^,^^22^ In this antibody chemiluminescent microparticle immunoassay (CMIA) test, the SARS-CoV-2 antigen coated paramagnetic microparticles bind to the IgG antibodies that attach to the viral nucleocapsid protein in human serum samples. The Sample/Calibrator index values of chemiluminescence in relative light units (RLU) of 1·40 (IgG assay), and 1·00 (IgM assay), respectively, and above were considered as positive per the manufacturer's instructions.

Quantitative detection of SARS-CoV-2 anti-S1/S2 IgG antibodies was performed using the DiaSorin LIAISON platform. The CLIA assay consists of paramagnetic microparticles coated with distally biotinylated S1 and S2 fragments of the viral surface spike protein. RLUs proportional to the sample's anti-S1/S2 IgG levels were converted to AU/mL based on a standardized master curve.

Semi-quantitative detection of SARS-CoV-2 anti-receptor binding domain (RBD) IgM antibodies was performed using the Wantai IgM-ELISA (CE-IVD) kit (Sanbio, Uden, The Netherlands).^23^ Briefly, the IgM µ-chain capture method was used to detect IgM antibodies using a double-antigen sandwich immunoassay using mammalian cell-expressed recombinant antigens containing the RBD of the spike protein of SARS-CoV-2 and the immobilized and horseradish peroxidase-conjugated antigen. Sample/Cut-off index OD values of 1 and higher were considered positive per the manufacturer's instructions.

Semi-quantitative detection of SARS-CoV-2 anti-S1 IgA antibodies was performed using the Euroimmun IgA 2-step ELISA.^24^ Ratio values of 1·1 and higher were considered positive per the manufacturer's instructions.

### Severity score calculation

The severity score is based on the 4C mortality score.^25^ The 4C mortality score is a prediction score calculated at admission, and the severity score calculated in our cohort represents the daily clinical disease severity, and thus is dependent on parameters that can change over time. Therefore, the fixed parameters of the 4C score were removed (i.e. age, sex at birth, number of comorbidities). Daily oxygen flow for non-ICU patients (L/min) and p/f ratio (kPa) and FiO_2_ (%) for ICU patients were added to our severity score **(Table S2)**. The severity score used in this analysis only applied to hospitalized patients. Since only hospitalized patients were included in the study, we did not include ‘hospitalization’ as a parameter in the score (as all patients would have similar points for hospitalization).

### Statistical analysis

**Figure 1:**
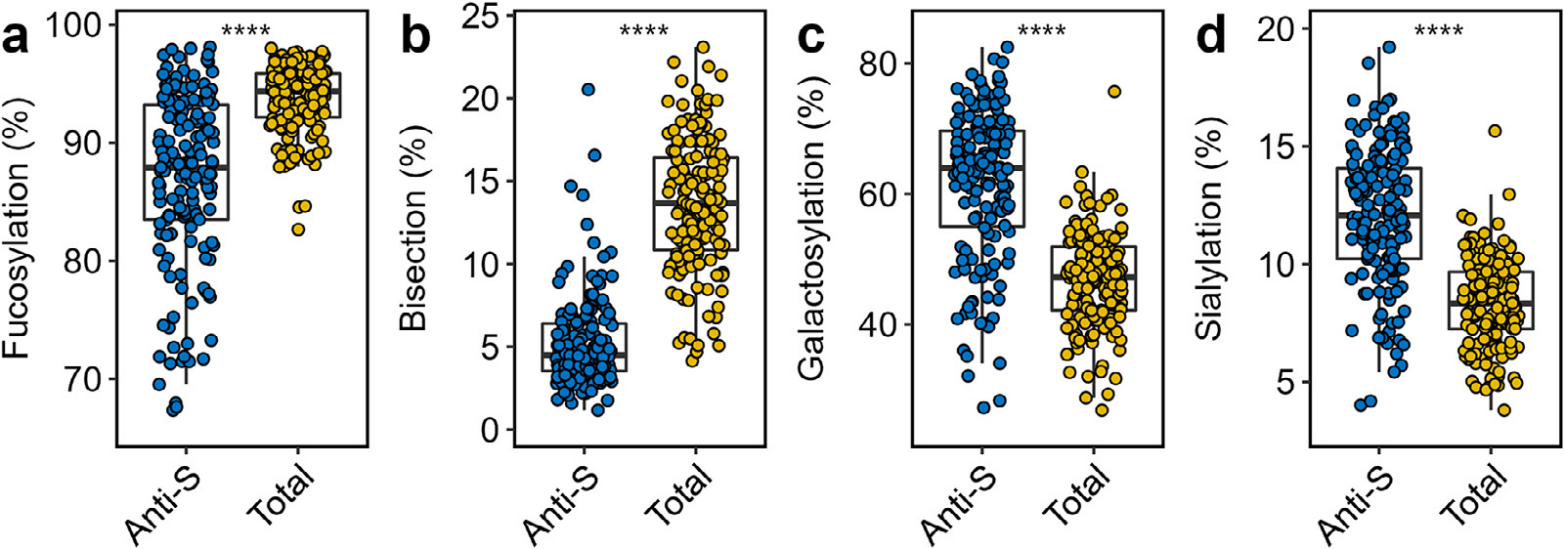
Comparison of anti-S (blue) and total (yellow) IgG1 Fc glycosylation. Relative abundance of IgG1 (a) fucosylation, (b) bisection, (c) galactosylation and (d) sialylation of anti-S and total IgG1 are given at hospitalization (n=159). Boxplots display the median and the interquartile range, whereas whiskers represent the first and third quartiles. A Wilcoxon signed-rank test was used to compare anti-S with total IgG1. ****: *p*-value < 0·0001. (For interpretation of the references to color in this figure legend, the reader is referred to the web version of this article.)

Relative intensity of each glycopeptide species in the final analyte list was calculated by normalizing to the sum of their total areas **(Table S3)**. Structurally similar glycopeptide species were used for the calculation of derived traits fucosylation, bisection, galactosylation and sialylation **(Table S4)**. Anti-S and total IgG1 glycosylation traits were compared using a Wilcoxon signed-rank test (Figure 1, Table S5), while a Wilcoxon rank-sum test was used to compare non-ICU and ICU patients as well as severity score and other groups (Figures. 3, 5**, S3, S7, S8, S10–13; Tables S6, S7, S9**). To account for multiple testing, *p*-values of the Wilcoxon-tests have been corrected by the Benjamini-Hochberg procedure to 5% FDR in each statistical question **(Tables S5–7, S9)**. Both anti-S and total IgG1 galactosylation were found to be confounded by age and sex **(Figure S2)** in line with literature on IgG Fc glycosylation.^26^ Therefore, delta (Δ) values were calculated by subtracting total from anti-S IgG1 levels to eliminate the confounding effect, which we believe was a sensible way of limiting the influence of possible other confounders as well **(Figs. S2, S3, S12, S13, Table S9)**. Receiver operating characteristics (ROC) were first assessed using all Δglycosylation traits with a significant difference between the ICU and non-ICU groups at time of hospitalization **(**Figure 4**)**. As only Δgalactosylation was found as a significant predictor of ICU admission, we decided not to show the composite model, but the individual ROC curves. The model was trained on a random selection of 70% of the patients’ samples, with the prediction being validated on the remaining 30% of the patients’ samples. Spearman's correlation was used to explore associations between Δglycosylation traits and age, and Δglycosylation traits and ICU admission and severity **(Figs. S2, S8, S11)**, as well as between Δglycosylation traits and inflammatory markers and titers **(**Figure 6**, Table S8)**. To assess method repeatability, the inter-plate variation for the 14 analytes included in the final analysis was calculated for the standards, which was 2·4%. All statistical analyses and visualizations were performed in R, version 4.1.0 (R Foundation for Statistical Computing, Vienna, Austria) and RStudio, version 1.4.1717 (RStudio, Boston, MA).

### Role of funding source

The funders had no role in study design, data collection, data analysis, data interpretation, or writing of the report.

## Results

Both anti-S and total IgG1 glycosylation signature of 159 COVID-19 patients and corresponding timepoints were analyzed during their entire hospitalization period. The patient demographics and the comprehensive cohort characteristics including comorbidities are presented in Table 1 and **Table S1**, respectively. Follow-up samples after hospital discharge were available for 19 patients **(Table S1, Figure S5)**. LC-MS was employed to analyze Fc glycosylation on the glycopeptide level after tryptic digestion, which allowed the identification of 14 glycoforms. The found glycoforms were consistent with previous reports on anti-S IgG1 glycosylation^13^^,^^14^, from which fucosylation, bisection, galactosylation and sialylation were calculated **(Tables S3 and S4)**. Overall, a total of 650 total IgG1 and 650 anti-S IgG1 glycosylation profiles were determined.

**Table 1:** Baseline patient characteristics. Median and interquartile ranges are shown unless indicated otherwise. The sex of one non-ICU patient is unknown.

	ICU (n=77)	non-ICU (n=82)
Age	65 (59-71)	66·5 (54-74·5)
Female, n (%)	18 (23)	21 (26)
Male, n (%)	59 (77)	60 (74)
Severity score	12 (10-14)	3 (2-4)
Days since symptom onset	15·5 (12-22)	13 (10-16)
BMI	29·1	26·8
Diabetes, n (%)	28 (36)	26 (31)

### Anti-S IgG1 Fc glycosylation of COVID-19 patients is skewed

The Fc glycosylation signatures of anti-S and total IgG1 were compared pairwise at hospitalization with regard to fucosylation, bisection, galactosylation and sialylation **(**Figure 1**, Table S5)**. Fucosylation of anti-S was significantly lower than total IgG1 (fold change (FC): 0·93; *p*-value: 3·4 × 10^−24^) **(**Figure 1**a, Table S5)**. Notably, a prominently low anti-S fucosylation (<85%) was found for 56 patients, with a few patients showing levels as low as 66% **(**Figure 1**a)**. Similarly, bisection of anti-S was markedly lower than total IgG1 (FC: 0·33; *p*-value: 3·1 × 10^−27^) **(**Figure 1**b)**. Anti-S galactosylation (FC: 1·35; *p*-value: 8·1 × 10^−26^) **(**Figure 1**c)** and sialylation (FC: 1·45; *p*-value: 2·7 × 10^−26^) **(**Figure 1**d)** were elevated as compared to their total IgG1 counterpart.

### Dynamic regulation of IgG1 Fc glycosylation in COVID-19

**Figure 2:**
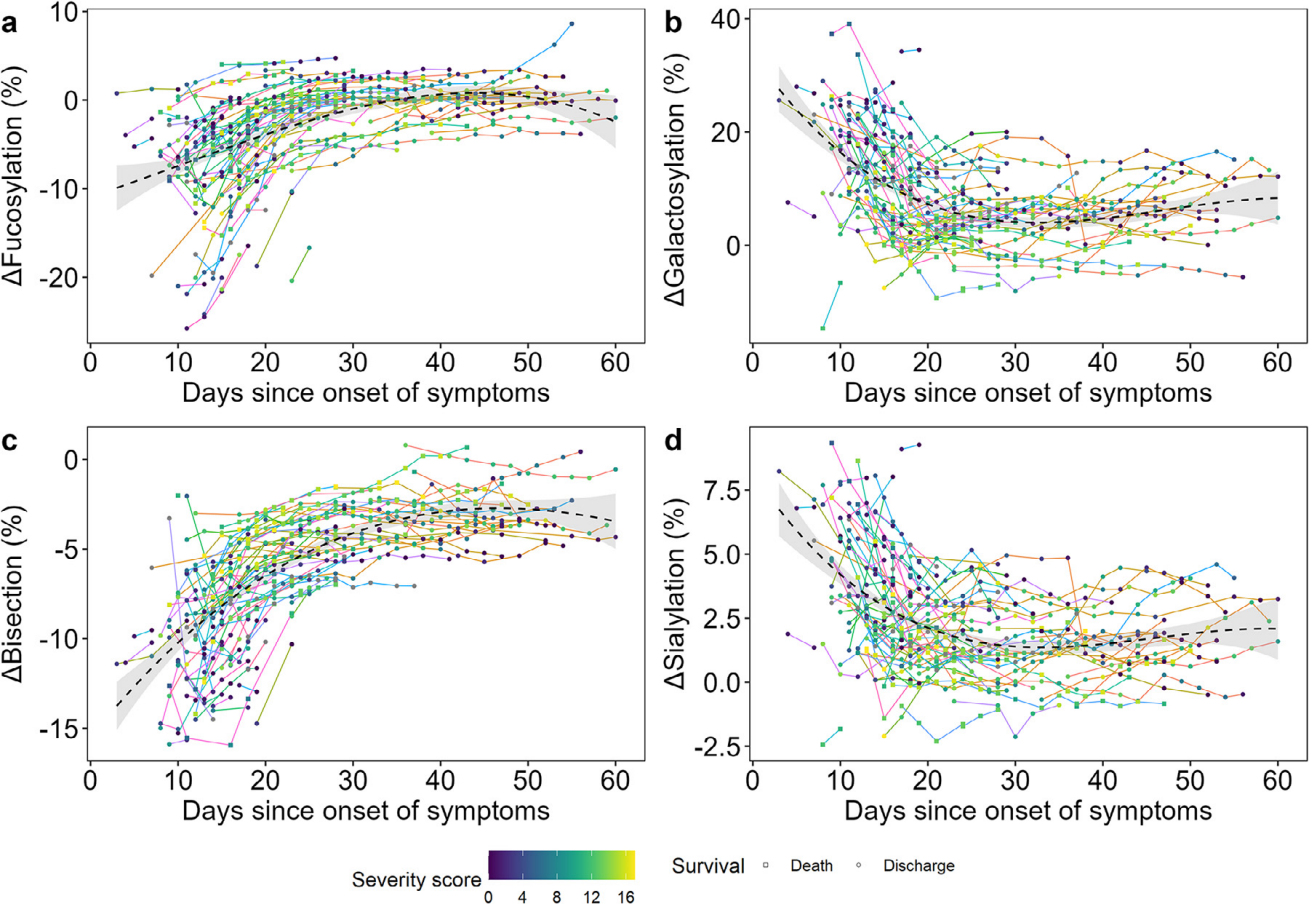
ΔGlycosylation dynamics until 60 days since symptom onset. The time course of Δglycosylation traits (a) fucosylation, (b) galactosylation, (c) bisection and (d) sialylation as shown during the hospitalization period (n=109). Line colors correspond to a single COVID-19 patient, whilst the color gradient in the circles/squares indicate the corresponding severity score (grey = NA). The shape displays whether a patient passed away (square) or was discharged alive (circle). The black dashed line with a grey 95% confidence interval band is a cubic polynomial fit over the shown datapoints to illustrate overall dynamics. Late timepoints and two outliers are shown in the Supplementary Material due to spatial constraints (Figs. S4 and S5), as well as anti-S and total IgG1 glycosylation dynamics (Figure S6).

Next, we explored the changes of glycosylation over time. Anti-S glycosylation was found to be highly dynamic, but also total IgG1 glycosylation showed changes in the course of the disease **(Figure S6**). The longitudinal samples allowed us to establish the time course of ΔIgG1 glycosylation during hospitalization, normalized for day of onset of symptoms **(**Figure 2**, Table S1)**. The Δ values were calculated by subtracting total from anti-S IgG1 levels, representing the skewing of anti-S as compared to total IgG1, and used hereafter. Interestingly, all glycosylation traits showed a transient pattern for most patients, and were characterized by profound dynamics, as illustrated by the timelines of individual patients (as indicated by differential line coloring) and by the fit cubic polynomial line **(**Figure 2**)**. Fucosylation **(**Figure 2**a)** and bisection **(**Figure 2**c)** showed a rapid increase within days and weeks after onset of the disease, followed by a plateau and approximation of the glycosylation patterns of total IgG1 **(Figure S6)**. In contrast, galactosylation **(**Figure 2**b)** and sialylation **(**Figure 2**d)** quickly declined in the first days and weeks, with the decrease continuing for a long period albeit at lower pace. At the moment of hospital discharge anti-S galactosylation and sialylation were still slightly higher than total IgG1. Since 19 convalescent patients returned for follow-up sampling after hospital discharge, we noted that for most, fucosylation and bisection largely remained constant or slightly increased, whilst galactosylation and sialylation continued to decrease since the last available timepoint **(Figure S5)**.

### IgG1 Fc glycosylation associates with ICU admission

**Figure 3:**
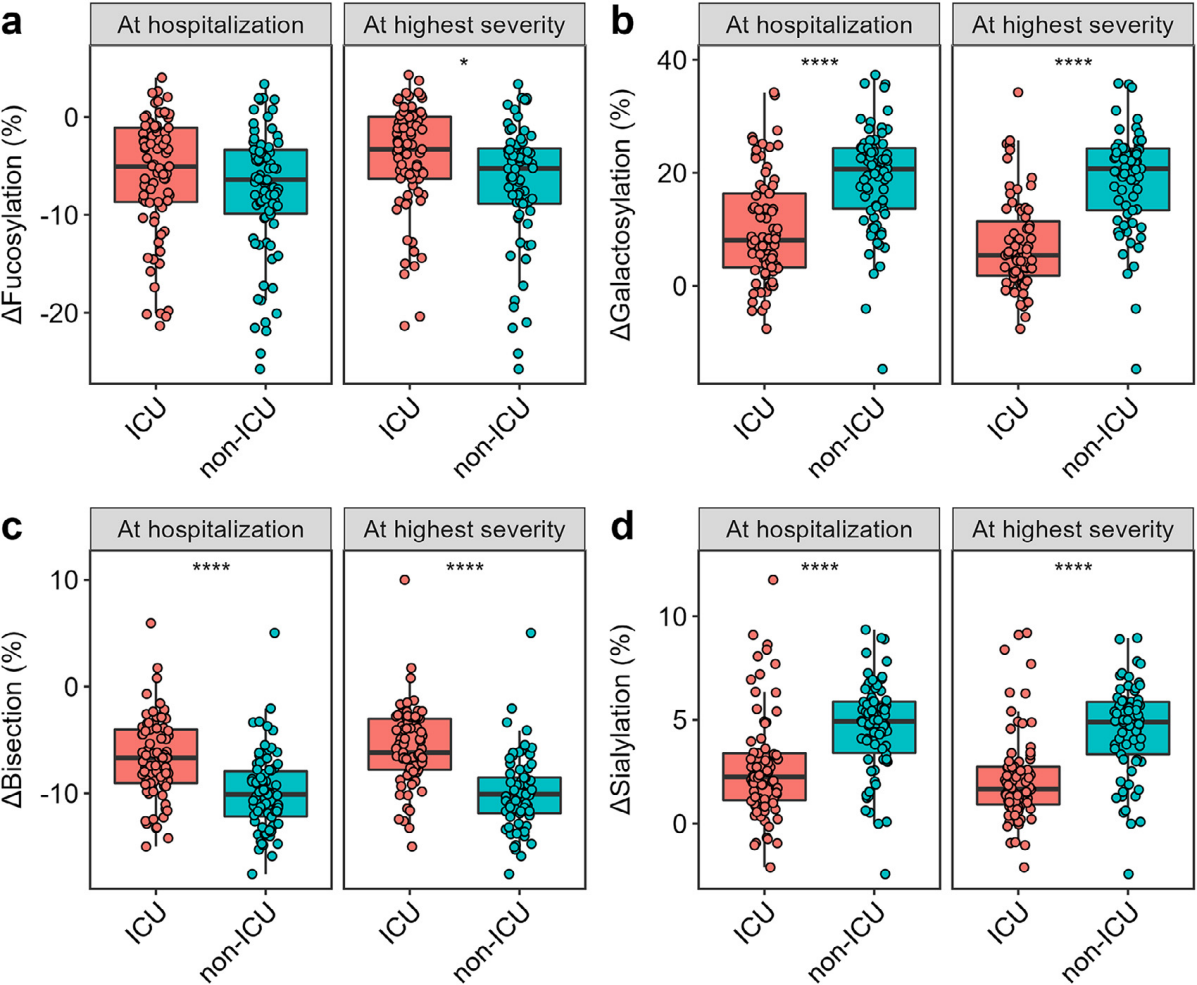
Comparison of Δglycosylation traits of patients admitted to ICU (red) or non-ICU (blue) treatment. Shown in the facets are the relative levels of ΔIgG1 (a) fucosylation, (b) galactosylation, (c) bisection and (d) sialylation at the time of hospitalization (left; n=159; 77 ICU and 82 non-ICU patients, respectively) and at the time of highest disease severity (right; n=144; 75 ICU and 69 non-ICU patients, respectively). The highest severity timepoint has been defined for each patient as the earliest possible timepoint with highest severity score during hospitalization. A Wilcoxon rank-sum test was used to compare ICU and non- ICU patients (Table S6). *, ****: *p*-value < 0·05, 0·0001, respectively. Glycosylation dynamics of ICU and non-ICU patients between day 10 and 25 are shown in Figure S8 (For interpretation of the references to color in this figure legend, the reader is referred to the web version of this article.).

To investigate whether Fc glycosylation was associated with intensive care unit (ICU) admission, patients were stratified based on treatment need. This resulted in two groups: (1) patients who at some point during hospitalization were admitted to the ICU, and (2) patients who were not enrolled to ICU treatment at all (non-ICU) during hospitalization. ΔIgG1 glycosylation derived traits fucosylation, bisection, galactosylation and sialylation of the above groups were compared both at time of hospitalization and at the time point of their highest disease severity **(**Figure 3**, Table S6)**.

ΔIgG1 Fc glycosylation of ICU patients showed a different profile from those of non-ICU patients, with the latter being characterized by lower bisection (FC: 0·66, *p*-value: 7·2 × 10^−8^) **(**Figure 3**c)**, and higher galactosylation (FC: 0·39, *p*-value: 2·9 × 10^−9^) **(**Figure 3**b)** and sialylation (FC: 0·46, *p*-value: 1·7 × 10^−7^) **(Figure 3d)** at the time of hospitalization. This difference was maintained or even more pronounced at the time of highest disease severity (FC: 0·61, 0·26, 0·34; *p*-value: 1·9 × 10^−10^, 4·1 × 10^−12^, 3·4 × 10^−9^), for Δbisection, Δgalactosylation and Δsialylation, respectively **(Table S6)**. ΔFucosylation levels of the ICU group were higher at the time of highest disease severity (FC 0·62; *p*-value: 0·012), but were similar at the time of hospital admission **(**Figure 3**a)**. The observed differences in Δgalactosylation and Δsialylation were reflected in changes of anti-S IgG1 glycosylation, while changes in Δbisection were largely attributed to alterations of total IgG1 levels **(Figure S7)**. To confirm that the observed effects were not confounded by vast glycosylation dynamics, a subset of non-ICU and ICU patients were created and compared, which resulted in comparable observations with regards to Δbisection, Δgalactosylation and Δsialylation as shown above **(Figs. S8 and S9)**.

**Figure 4:**
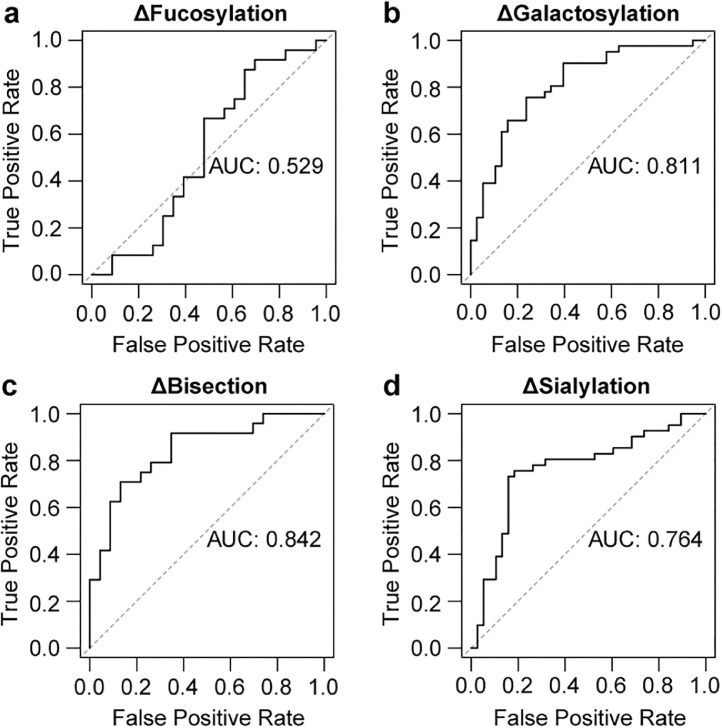
ROC curves and corresponding AUC values illustrating the power of certain ΔIgG1 glycosylation traits to predict ICU admission at time of hospitalization.

Based on the found associations, ROC curves were generated using the baseline timepoints (Figure 4**)**, which illustrated the discriminative potential of **Δ**galactosylation (area under the curve (AUC): 0.811) and **Δ**bisection (AUC: 0.842) for ICU admission.

### IgG1 Fc glycosylation associates with disease severity

**Figure 5:**
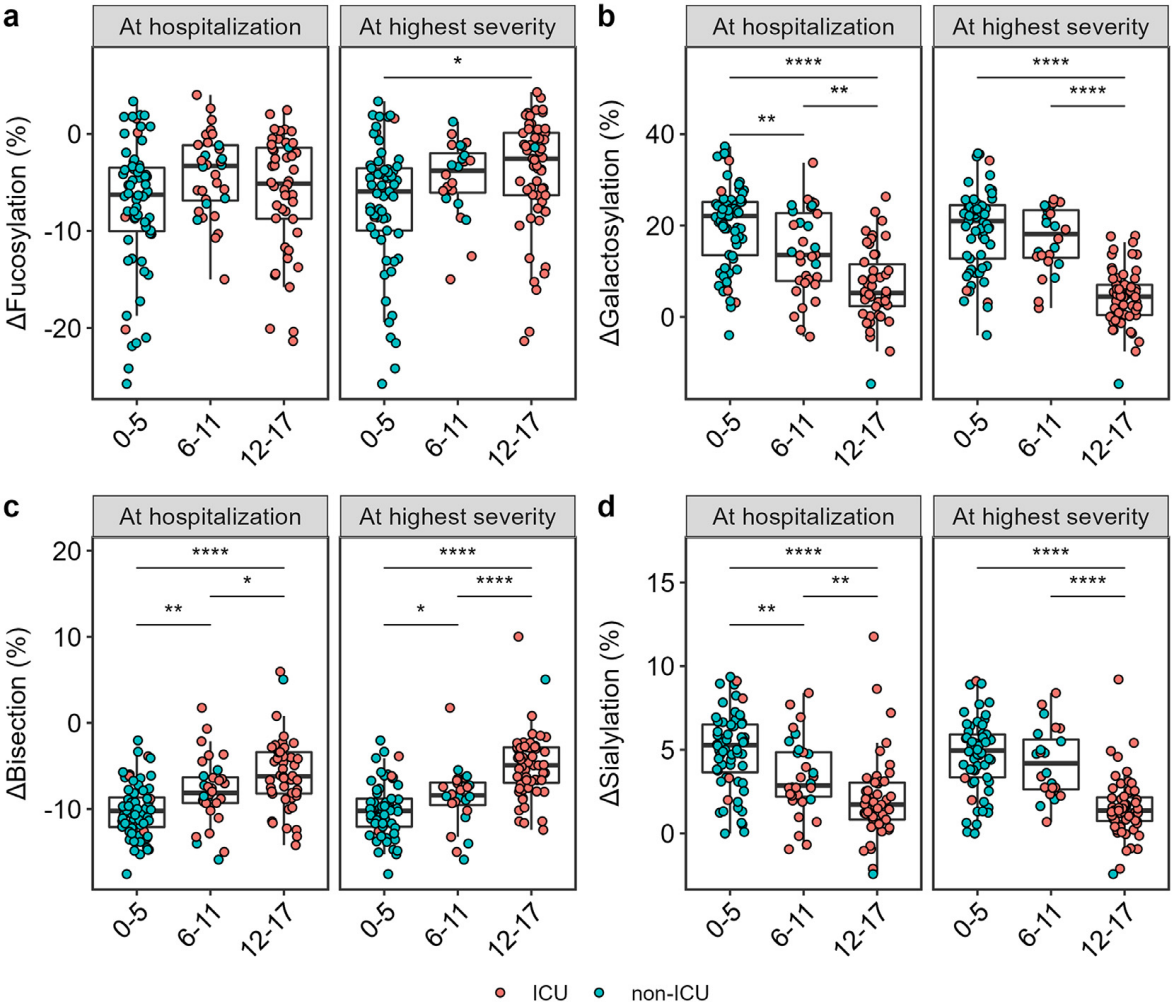
Comparison of Δglycosylation of patients in different severity score groups. Shown in the facets are the relative levels of ΔIgG1 (a) fucosylation, (b) galactosylation, (c) bisection and (d) sialylation at the time of hospitalization (left; n=142; 64 low severity, 32 intermediate severity and 46 high severity patients, respectively) and at the time of highest disease severity (right; n=144 n=144; 61 low severity, 24 intermediate severity and 59 high severity patients, respectively). Color indicates ICU (red) and non-ICU (blue) patients. A Wilcoxon rank-sum test was used to compare the different severity score groups (Table S7). *, **, ****: *p*-value < 0·05, 0·01, 0·0001, respectively (For interpretation of the references to color in this figure legend, the reader is referred to the web version of this article.).

Patients were stratified into three groups based on their severity score: (1) severity score between 0 and 5 (low severity), (2) 6–11 (intermediate severity) and (3) 12–17 (high severity). Similarly as before, ΔIgG1 glycosylation traits were compared both at time of hospitalization and at time of highest disease severity **(**Figure 5**, Table S7)**.

ΔBisection was found to be increased in groups with increased disease severity **(**Figure 5**c)**, whereas Δgalactosylation **(**Figure 5**b)** and Δsialylation **(**Figure 5**d)** patterns were found to be decreased with increased disease severity at the time of hospitalization **(Table S7)**. These observations were largely maintained at highest disease severity **(**Figure 5**, Table S7)**. Higher fucosylation marked the time of highest disease severity, but remained rather stable at the time of hospital admission between all groups **(**Figure 5**a, Table S7)**. The observed differences in Δgalactosylation and Δsialylation reflecting in changes of anti-S IgG1 glycosylation, while changes in Δbisection were largely attributed to alterations of total IgG1 levels **(Figure S10)**. To confirm that the observed effects were not confounded due to profound glycosylation dynamics, subsets of patients matched for the time since disease onset were compared, which resulted in similar observations with regards to Δgalactosylation and Δsialylation as shown above, whereas we could not exclude a potential confounding effect for the Δbisection signature, maybe caused by swift glycosylation dynamics, low sample size, or the combination thereof **(Figure S11)**. Apart from ICU admission and severity score, we tested acute respiratory syndrome, ventilation, survival, diabetes and BMI, and found Δbisection being higher for patients at baseline who passed away later, but no other associations were found **(Figs. S12 and S13)**.

### IgG1 Fc glycosylation associates with inflammatory markers

**Figure 6:**
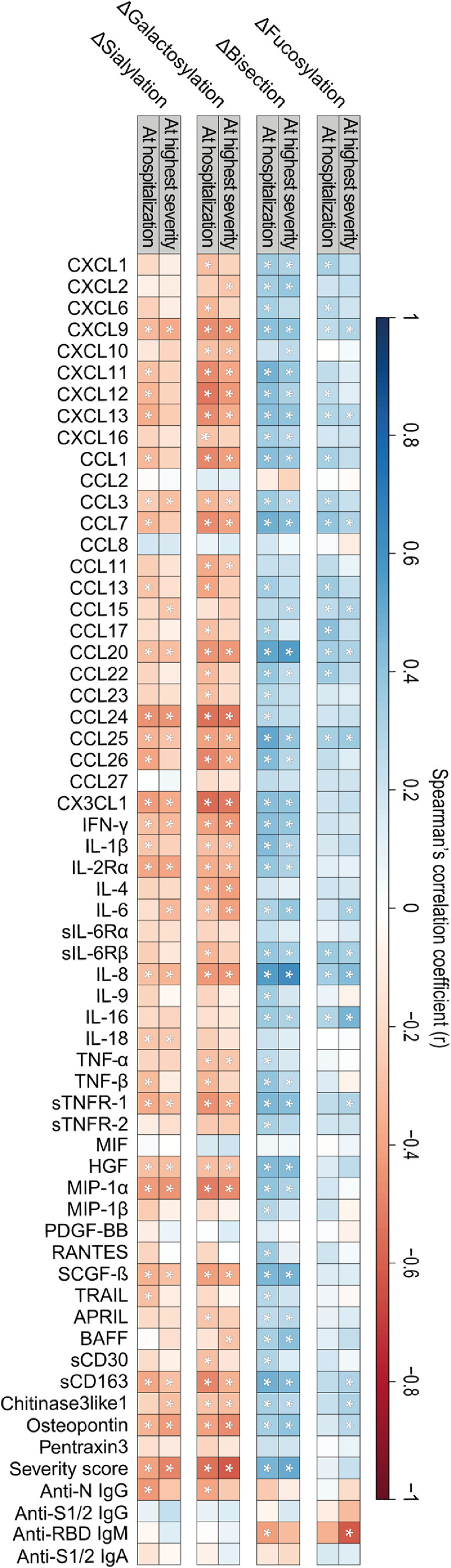
Heatmap visualizing Spearman's correlations between Δglycosylation traits and inflammatory markers at time of hospitalization (left side of each panel; n=58) and at time of highest disease severity (right side of each panel; n=59). Asterisk (*) indicates a significant Spearman's correlation (*p*-value < 0.05).

Multiple inflammatory mediators (in serum) and clinical parameters were measured for patients enrolled during the first wave of the pandemic. These include members of the CXC, CCL and CX3C chemokine families, cytokines and corresponding soluble receptors, acute phase proteins and other mediators involved in the immune response as well as severity scores and anti-viral antibody titers. In general, negative associations were found between Δgalactosylation and Δsialylation and positive associations for Δbisection and Δfucosylation with inflammatory markers at baseline. One notable exception was a strong negative correlation between anti-RBD IgM levels and Δbisection and Δfucosylation at baseline and at highest severity, respectively. ΔSialylation associated negatively with various chemokines, such as CCL24 (r = -0·45), CX3CL1 (r = -0·43), CCL25 (r = -0·34), certain cytokines, such as IL-8 (r = -0·29), IFN-γ (r = -0·3) and several other variables **(**Figure 6**, Table S8)**.

Comparable, and largely overlapping negative associations were found for Δgalactosylation as for Δsialylation: CCL24 (r = -0·55), CX3CL1 (r = -0·56), CCL25 (r = -0·41), IL-8 (r = -0·44), INF-γ (r = -0·4) and TNF-β (r = -0·33). Conversely, Δbisection associated positively with IL-8 (r = 0·56), CCL25 (r = 0·52) and CX3CL1 (r = 0·56). Additionally, severity score negatively correlated with Δgalactosylation (r = -0·55) and Δsialylation (r = -0·41) and positively with Δbisection (r = 0·46). Positive associations were found between Δfucosylation and inflammatory markers, including CCL17 (r = 0·41) and IL-8 (r = 0·34). The above described baseline correlations were comparable to those at the time of highest disease severity, but a vast body of associations were temporary **(**Figure 6**, Table S8)**. Interdependencies of the various IgG glycosylation traits have been described^18^ and as well observed in this study, which are reported in **Table S8**.

## Discussion

In this study, we analyzed total and anti-S IgG1 Fc glycosylation of 159 COVID-19 patients at different timepoints during their clinical illness. Although several studies reported on the importance of (anti-S) IgG1 Fc glycosylation and its association with disease severity in COVID-19,^27^ this study involves a large, single center cohort that confirms specific anti-S IgG1 glycosylation features as an early hallmark of severe COVID-19 in an age- and sex-corrected, time-matched dataset at baseline, and in the longitudinal dimension.

Afucosylated IgG1 B cell responses have recently been described to characterize immune reactions against membrane-embedded antigens in general, and in particular against viral infections caused by enveloped viruses such as COVID-19.^13^ Foregoing studies showed that severe, hospitalized patients exhibit a decreased anti-S IgG1 fucosylation as compared to mild, non-hospitalized patients.^6^^,^^13^^,^^14^ Accordingly, we likewise observed proinflammatory, low-fucosylation signatures on anti-S as compared to total IgG1, but found no difference in fucosylation comparing hospitalized ICU patients versus hospitalized non-ICU patients, which is in line with a previous report on anti-SARS-CoV-2 RBD IgG1 fucosylation.^14^ Therefore, based on the early existence of these proinflammatory signatures in some of the patients, we hypothesize that low fucosylation – potentially even lower before measurable seroconversion, as hypothesized before^13^ – on anti-S IgG1 may act as an early inflammatory signal that promotes the development of a more severe disease in COVID-19 patients, resulting in hospital admission. However, disease severity between hospitalized patients could not be further distinguished based on anti-S IgG1 fucosylation. Furthermore, hardly any negative associations were found between anti-S IgG1 fucosylation and inflammatory markers in this study, unlike in previous reports, where *in vitro* experiments demonstrated that the stimulation of isolated macrophages with recombinant, glycoengineered anti-S or patient sera-derived low-fucose IgG1 antibodies trigger higher proinflammatory cytokine release than those with normal fucose levels.^6^^,^^13^^,^^14^ However, high proinflammatory cytokine levels are not necessarily present in all severe patients,^28^ and this contrasting observation suggests a different regulation and/or the temporal resolution of fucosylation and cytokine production dynamics *in vivo*. Additionally, beyond or in combination with low anti-S IgG1 fucosylation a pre-existing risk factor may play a role in COVID-19 disease severity, which hitherto remained unclear.^29^ Of note, the anti-S and anti-RBD IgG1 Fc glycosylation data were all determined from the circulation, and it is unclear to which extent this would reflect the inflammatory pattern and glycosylation profile of anti-S antibodies in the lung. Our results demonstrate that the proinflammatory fucosylation signature that is observed at the early time points in the disease tends to fade with the course of the disease, which one may interpret as a shift towards a more anti-inflammatory Fc glycosylation profile that is maintained over time. The absence of core fucose is known to enhance a proinflammatory immune response by activating FcγRIII receptors on monocytes, macrophages and NK cells.^10^ Decreased fucosylation on specific IgG1 has been described in HIV^13^^,^^30^ and dengue fever,^31^ as well as in alloimmune diseases.^32^, ^33^, ^34^, ^35^, ^36^ However, whilst afucosylation of specific IgG1 plays a protective role in HIV, it clearly marks high disease severity in dengue, alloimmune diseases or COVID-19.^6^^,^^13^^,^^14^ Furthermore, low total IgG1 fucosylation has been associated with outcome of pediatric meningococcal sepsis indicating a systemic inflammation due to the potential accumulation of airway infections during early childhood.^37^ Even though the origin of low fucose IgG responses is seemingly linked to antigen context and affect mostly specific antibodies,^13^ the mechanisms underlying the dynamics of antibody glycosylation remain elusive.

Besides afucosylation, a transient, decreased bisection was found on anti-S IgG1. Recent reports suggest that severe COVID-19 patients present low levels of bisection both on total IgG (Fc and Fab combined)^29^ and anti-S IgG1^13^ relative to mild cases. In contrast, no difference was found in anti-RBD IgG1 bisection between ICU and non-ICU patients in age- and sex-matched patients,^14^ albeit these disease groups were largely comparable to the ones in our study. While bisection associated positively with, and was an important predictor for ICU admission, disease severity and survival in our study, it lacks functional relevance based on our current understanding and has no effect on FcγRIII or C1q binding.^10^^,^^38^

Elevated galactosylation and sialylation of anti-S IgG1 were associated with a less severe disease course upon hospitalization, and no ICU admission. Similar observations were made in a previous report, where severe COVID-19 was characterized by lower anti-S IgG1 galactosylation and sialylation than mild COVID-19.^13^ Interestingly, both anti-S and total IgG1 galactosylation and sialylation decrease by advancing age. As Larsen et al. compared anti-S IgG1 galactosylation and sialylation of imperfectly age matched patient groups without age correction, the disease and age effects remained indiscernible.^13^ We describe decreased anti-S IgG1 galactosylation in ICU patients as compared to non-ICU patients, and analogously, markedly lower specific IgG1 galactosylation has been shown to characterize the more severe, active phase of tuberculosis as compared to its latent counterpart.^39^ Even though more and more reports support that elevated levels of galactosylated IgG are associated with the activation of the classical complement pathway^10^^,^^12^^,^^40^, agalactosylation was associated with increased disease severity in this study, possibly due to the fact that complement can contribute to the increased inflammation both directly, and through inducing a chemotactic response through C5a, thereby increasing cellular infiltration to inflamed tissues such as the lung.^41^ Furthermore, galactosylation appeared to be another predictor for ICU admission besides bisection. Elevated sialylation levels on anti-S IgG1 were associated with low disease severity in the current report. Sialylation has been broadly described as critical in mediating anti-inflammatory activity,^42^, ^43^, ^44^ yet it remains to be elucidated whether sialylated IgG exerts an anti-inflammatory effect in COVID-19.

The study has a number of limitations. Firstly, this study reports on the results obtained in a single center cohort study where patient numbers were limited by availability. Therefore, the results may to some extent be influenced by small sample size. Secondly, demographic characteristics and comorbidities, in particular age, sex, BMI and diabetes are known confounders of IgG glycosylation. Although such associations were not revealed by the performed statistical tests, these features, together with other unknown factors may led to uncertainty in study inferences. As a third limitation, while the study points to a possible link between IgG1 Fc glycosylation and disease mechanism, supporting functional and mechanistic data is missing.

## Conclusions

This study established anti-S IgG1 bisection, galactosylation and sialylation as a unique combination of features that associate with ICU admission and disease severity in hospitalized COVID-19 patients. These features were additionally associated with markers of inflammation and showed discriminative potential based on ROC assessment. Further studies, involving a larger study population are needed to see whether anti-S IgG1 glycosylation, in combination with other inflammatory markers, may be of value for patient stratification upon hospitalization. The glycosylation profiles are highly dynamic, the drivers of which remain elusive and to be investigated in future studies.

## Contributors

T. P.: Data (pre)processing, data curation, formal analysis, validation, investigation, visualization, statistical analysis, data interpretation, conceptualization, writing – original draft preparation. J. N.: sample preparation, data acquisition (IgG Fc glycosylation), W. W.: sample preparation (IgG Fc glycosylation), F. L.: production and purification of recombinant spike protein, K. E. van M, S. A. J., T. H. M. O: data acquisition (soluble marker profiles), review J. J. C. V.: data acquisition (antibody titers), writing – review & editing, A. H. E. R., S. M. A.: set up of cohort, recruitment & sampling of participants, writing – review & editing, G.V., C. H. H.: writing – reviewing & editing M. W.: Supervision, writing – review & editing, conceptualization, funding acquisition.

All authors were involved in the critical revision of the manuscript and have given approval to the final version of the manuscript. T. P. and M. W. verified the underlying data.

## Declaration of interests

A. H. E. R received support from Crowdfunding Wake Up To Corona, organized by the Leiden University Fund, participated in grants or contracts with Diorapthe, Stichting apothekers and UNeedle, participated on a Data Safety Monitoring/Advisory Board of a multicenter Dutch clinical trial (Clinical trial (RCT) on convalescent plasma for treatment of immunocompromised patients with COVID-19) and has recently been appointed as member of the EMA scientific advisory group on vaccines (unpaid).

The other authors declare that the research was conducted in the absence of any commercial or financial relationships that could be construed as a potential conflict of interest.

## Appendix Supplementary materials

Image, application 1

Image, application 2
