# Supplementary material for: Immunoglobulin G1 Fc glycosylation as an early hallmark of severe COVID-19
Source: eBioMedicine. 2022 Mar 22;78:103957. doi: 10.1016/j.ebiom.2022.103957 (PMC8938159; doi:10.1016/j.ebiom.2022.103957)
Supplement: Supplementary file 1 [file mmc1.pdf]

SUPPORTING INFORMATION

EBioMedicine

Immunoglobulin G1 Fc glycosylation as an early hallmark of severe COVID-19

Tamas Pongracz[1,*], Jan Nouta[1], Wenjun Wang[1], Krista E. van Meijgaarden[4], Federica Linty[2,3], Gestur Vidarsson[2,3], Simone A. Joosten[4], Tom H. M. Ottenhoff[4], Cornelis H. Hokke[5], Jutte J. C. de Vries[6], Sesmu M. Arbous[7], Anna H. E. Roukens[7], Manfred Wuhrer[1] in collaboration with BEAT-COVID[#] and COVID-19[&] groups

[1]Center for Proteomics and Metabolomics, Leiden University Medical Center, Leiden, Netherlands
[2]Dept. of Experimental Immunohematology, Sanquin Research, Amsterdam, Netherlands
[3]Landsteiner Laboratory, Amsterdam University Medical Center, Amsterdam, Netherlands
[4]Dept. of Infectious Diseases, Leiden University Medical Center, Leiden, Netherlands
[5]Dept. of Parasitology, Leiden University Medical Center, Leiden, Netherlands
[6]Dept. of Medical Microbiology, Leiden University Medical Center, Leiden, Netherlands
[7]Dept. of Intensive Care, Leiden University Medical Center, Leiden, Netherlands

[#]**BEAT-COVID group** (in alphabetical order, investigators): B. M. van den Berg[2], S. Cannegieter[3], C. M. Cobbaert[4], A. van der Does[5], J. J. M. van Dongen[6], H. C. J. Eikenboom[7], M. C. M. Feltkamp[8], A. Geluk[9], J. J. Goeman[10], M. Giera[11], T. Hankemeier[12], M. H. M. Heemskerk[13], P. S. Hiemstra[5], J. J. Janse[14], S. P. Jochems[14], M. Kikkert[8], L. Lamont[12], J. Manniën[10], M. R. del Prado[1], N. Queralt Rosinach[15], M. Roestenberg[14], M. Roos[15], H. H. Smits[14], E. J. Snijder[8], F. J. T. Staal[6], L. A. Trouw[6], R. Tsonaka[10], A. Verhoeven[11], L. G. Visser[9], J. J. C. de Vries[8], D. J. van Westerloo[1], J. Wigbers[1], H. J. van der Wijk[10], R. C. van Wissen[4], M. Yazdanbakhsh[14], M. Zlei[6]

[1]Dept. of Intensive Care, Leiden University Medical Center, Leiden, Netherlands
[2]Dept. of Internal Medicine, Nephrology, Leiden University Medical Center, Leiden, Netherlands
[2]Dept. of Clinical Epidemiology, Leiden University Medical Center, Leiden, Netherlands
[4]Dept. of Clinical Chemistry, Leiden University Medical Center, Leiden, Netherlands
[5]Dept. of Pulmonary Medicine, Leiden University Medical Center, Leiden, Netherlands
[6]Dept. of Immunology, Leiden University Medical Center, Leiden, Netherlands
[7]Dept. of Internal Medicine, Thrombosis and Hemostasis, Leiden University Medical Center, Leiden, Netherlands
[8]Dept. of Medical Microbiology, Leiden University Medical Center, Leiden, Netherlands
[9]Dept. of Infectious Diseases, Leiden University Medical Center, Leiden, Netherlands
[10]Dept. of Biomedical Data Sciences, Leiden University Medical Center, Leiden, Netherlands
[11]Center for Proteomics and Metabolomics, Leiden University Medical Center, Leiden, Netherlands
[12]Dept. of Analytical Biosciences, Leiden Academic Centre for Drug Research, Leiden, Netherlands
[13]Dept. of Hematology, Leiden University Medical Center, Leiden, Netherlands
[14]Dept. of Parasitology, Leiden University Medical Center, Leiden, Netherlands
[15]Dept. of Human Genetics, Leiden University Medical Center, Leiden, Netherlands

[&]**COVID-19 group** (in alphabetical order, investigators)**:**
M. Baysan[2,3], M. G. J. de Boer[4], A. G. van der Bom[3], O. M. Dekkers[3], A. M. Eikenboom[3], S. B. ter Haar[3], L. Heerdink[3], L. J. van Heurn[3], I. de Jonge[3], W. Lijfering[3], R. Meier[1], J. A. Oud[1], F. Rosendaal[3], A. G. L. Toppenberg[3], J. Uzorka[4], A. A. van IJlzinga Veenstra, J. Wigbers[2], J. M. Wubbolts[4]

[1]Dept. of Hematology, Leiden University Medical Center, Leiden, Netherlands
[2]Dept. of Intensive Care, Leiden University Medical Center, Leiden, Netherlands
[3]Dept. of Clinical Epidemiology, Leiden University Medical Center, Leiden, Netherlands
[4]Dept. of Infectious Diseases, Leiden University Medical Center, Leiden, Netherlands

[*]**Correspondence:**
Tamas Pongracz

Albinusdreef 2, 2223ZA, Leiden, Netherlands

Tel: +31 71 526 8701
t.pongracz@lumc.nl

Table of contents

Supplementary Fig. 1. Age and sex distribution in the BEAT-COVID cohort.

Supplementary Fig. 2. Anti-S (blue) and total (yellow) IgG1 bisection, galactosylation and sialylation are confounded by age in a similar way, which is eliminated by normalizing to total IgG1 levels.

Supplementary Fig. 3. Comparison of Δglycosylation traits of female and male patients demonstrates the absence of a sex confounding effect.

Supplementary Fig. 4. ΔIgG1 glycosylation dynamics during the entire hospitalization period.

Supplementary Fig. 5. ΔIgG1 glycosylation dynamics of the subset of patients with a follow-up sample.

Supplementary Fig. 6. Anti-S and total IgG1 glycosylation dynamics during the entire hospitalization period.

Supplementary Fig. 7. Comparison of total (a, c, e, g) and anti-S (b, d, f, h) IgG1 glycosylation traits as per ICU admission.

Supplementary Fig. 8. ICU (red) and non-ICU (blue) patients and corresponding ΔIgG1 glycosylation derived traits in a "days since onset of symptoms" subset of patients (n=129) to confirm that the observed differences (Fig. 3) are not confounded by vast glycosylation dynamics.

Supplementary Fig. 9. ΔIgG1 Δglycosylation dynamics of patients admitted to the ICU (n=48; red) and non-ICU (n=34; blue) treatments as shown between days 10 and 25.

Supplementary Fig. 10. Comparison of total (a, c, e, g) and anti-S (b, d, f, h) IgG1 glycosylation traits as per severity score group.

Supplementary Fig. 11. Patients in varying severity score groups 0-5 (red), 6-11 (green) and 12-17 (dark blue) and corresponding ΔIgG glycosylation derived traits in a "days since onset of symptoms" subset of patients to confirm that the observed differences (Fig. 4) are not confounded by vast glycosylation dynamics.

Supplementary Fig. 12. Comparison of acute respiratory distress syndrome (a-d), survival (e-h) and ventilation (i-l), diabetes (m-p) subgroups and BMI (q-t) of patients for glycosylation traits fucosylation (a, e, i, m, q), bisection (b, f, j, n, r), galactosylation (c, g, k, o, s) and sialylation (d, h, l, p, t).

Supplementary Fig. 13. Comparison of diabetes (a-h) subgroups and BMI (i-o) of patients for total and anti-S IgG1 glycosylation traits fucosylation (a, e, i, m), bisection (b, f, j, n), galactosylation (c, g, k, o) and sialylation (d, h, l, p).

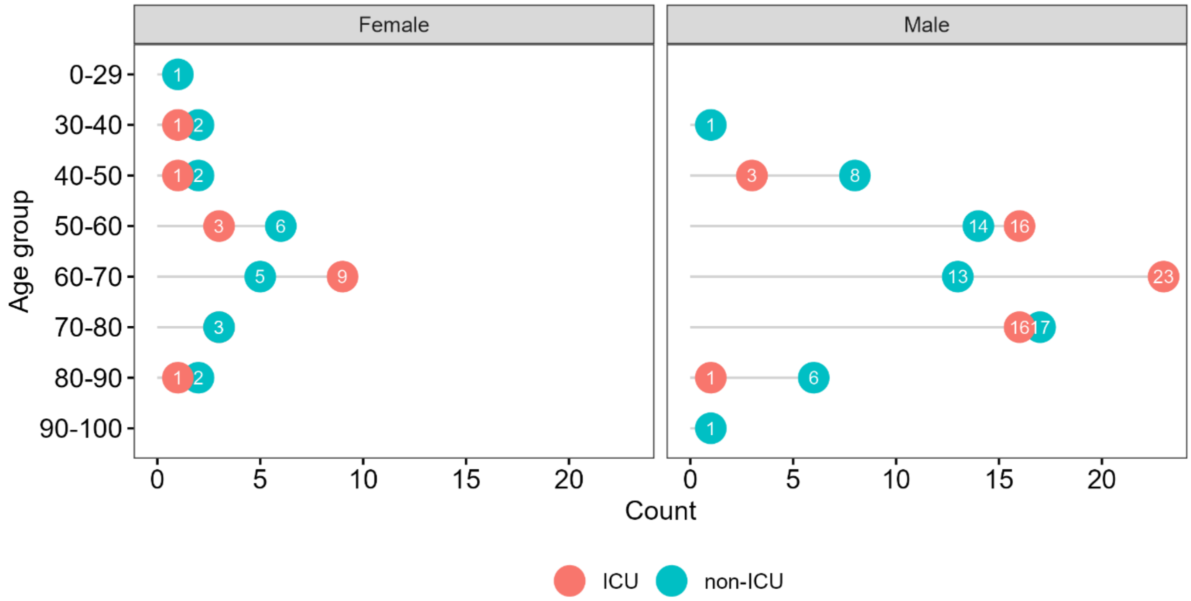

Supplementary Fig. 1. Age and sex distribution in the BEAT-COVID cohort. Overall 159 patients participated in the study (39 female and 119 male, 1 unknown (not shown)). The color illustrates ICU (red) and non-ICU (blue) treatment groups, whereas the number in the circles indicates the number of patients in the corresponding group.

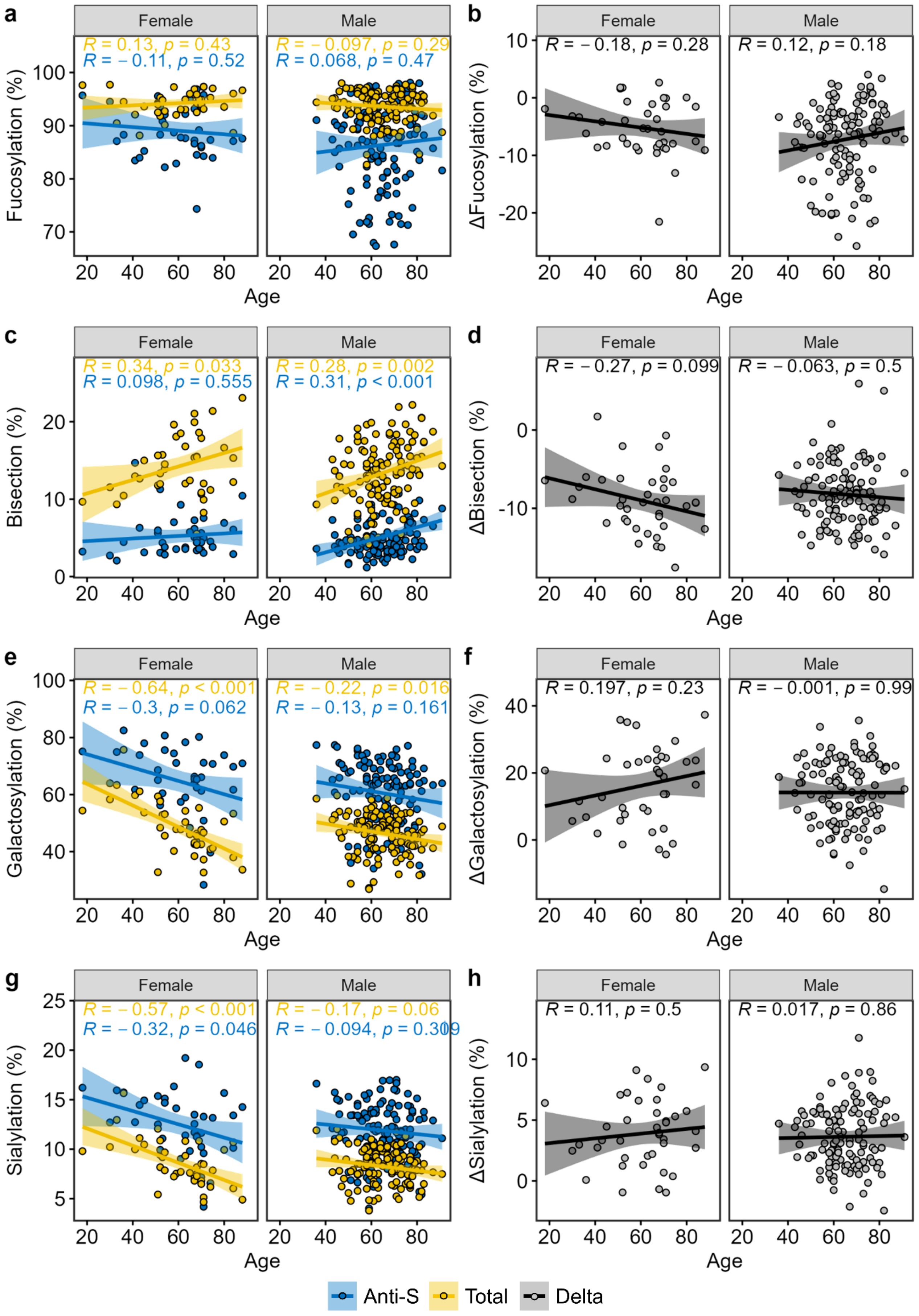
a
Female
Male
R = 0.13, p = 0.43
R = − 0.11, p = 0.52
R = − 0.097, p = 0.29
R = 0.068, p = 0.47
Fucosylation (%)
Age
b
Female
Male
R = − 0.18, p = 0.28
R = 0.12, p = 0.18
ΔFucosylation (%)
Age
c
R = 0.34, p = 0.033
R = 0.098, p = 0.555
R = 0.28, p = 0.002
R = 0.31, p < 0.001
Bisection (%)
Age
d
R = − 0.27, p = 0.099
R = − 0.063, p = 0.5
ΔBisection (%)
Age
e
R = − 0.64, p < 0.001
R = − 0.3, p = 0.062
R = − 0.22, p = 0.016
R = − 0.13, p = 0.161
Galactosylation (%)
Age
f
R = 0.197, p = 0.23
R = − 0.001, p = 0.99
ΔGalactosylation (%)
Age
g
R = − 0.57, p < 0.001
R = − 0.32, p = 0.046
R = − 0.17, p = 0.06
R = − 0.094, p = 0.309
Sialylation (%)
Age
h
R = 0.11, p = 0.5
R = 0.017, p = 0.86
ΔSialylation (%)
Age
Anti-S
Total
Delta

Supplementary Fig. 2. Anti-S (blue) and total (yellow) IgG1 bisection, galactosylation and sialylation are confounded by age in a similar way, which is eliminated by normalizing to total IgG1 levels. IgG1 **(a)** fucosylation, **(c)** bisection, **(e)** galactosylation and **(g)** sialylation as a proxy of age in female (left) and male (right) patients. Corresponding delta (Δ) IgG1 **(b)** fucosylation and **(d)** bisection, **(f)** galactosylation and **(h)** sialylation levels (all in grey), as normalized to total IgG levels by subtracting total from anti-S IgG1 glycosylation levels. Baseline timepoints are shown. Shown in the inset are the Spearmen correlation coefficients (R) and *p*-values, respectively. IgG1 bisection is known to increase, whereas galactosylation and sialylation are known to decrease upon aging.[1] Correction for the age confounding effect was performed by normalizing to total IgG levels, as illustrated by the weak and non-significant Spearman correlations and *p*-values, respectively **(b, d, f, h)**.

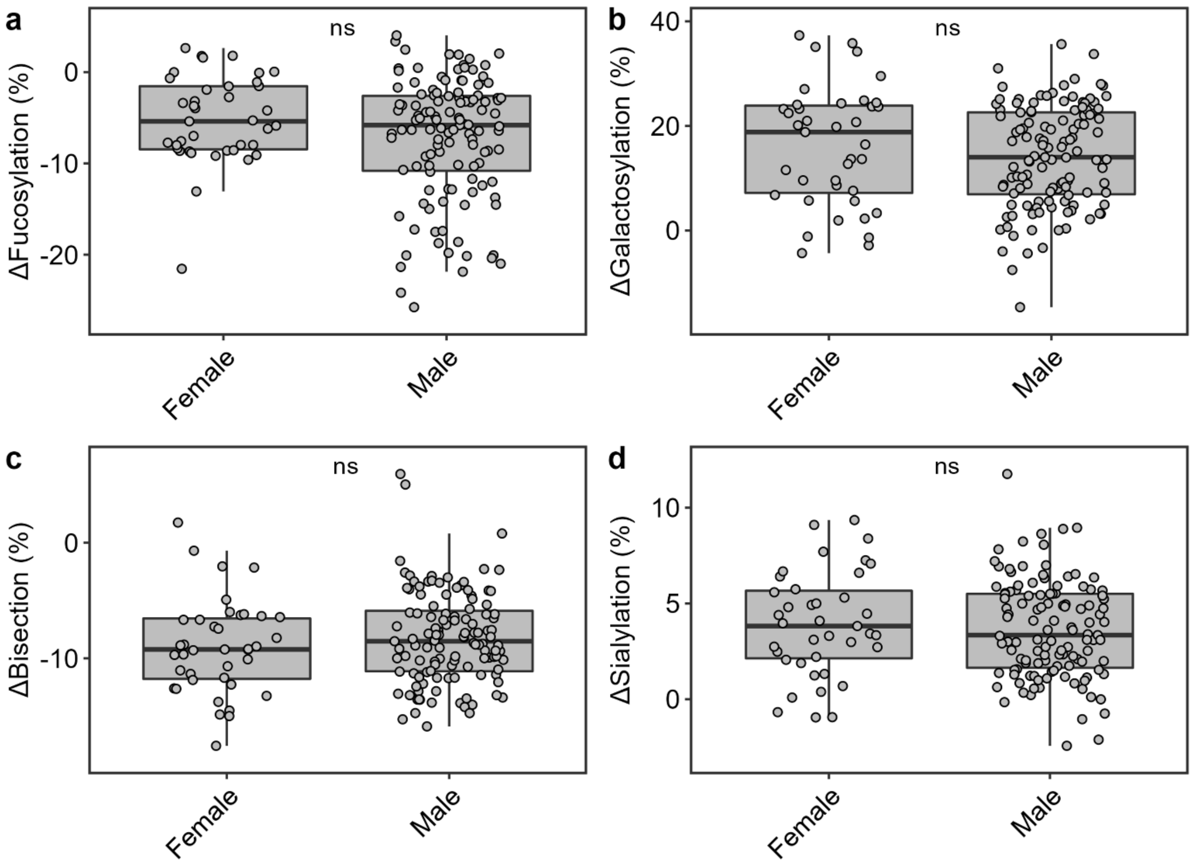

Supplementary Fig. 3. Comparison of Δglycosylation traits of female and male patients demonstrates the absence of a sex confounding effect. IgG1 **(a)** Δfucosylation, **(b)** Δgalactosylation, **(c)** Δbisection and **(d)** Δsialylation. Correction for the age and sex confounding effect was performed as described above **(Fig. S2)**.

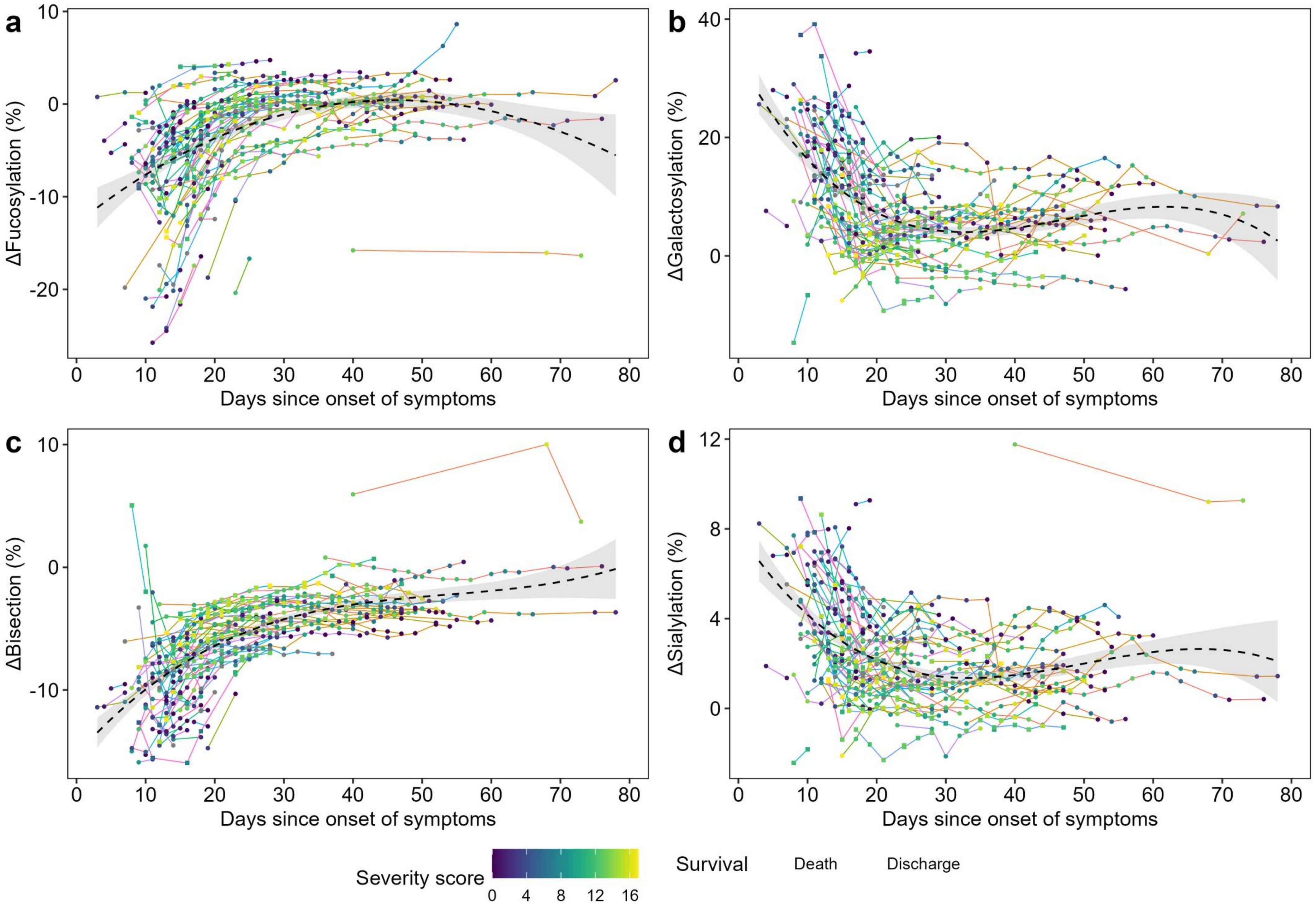
a
b
c
d
ΔFucosylation (%)
ΔGalactosylation (%)
ΔBisection (%)
ΔSialylation (%)
Days since onset of symptoms
Severity score
0
4
8
12
16
Survival
Death
Discharge

Supplementary Fig. 4. ΔIgG1 glycosylation dynamics during the entire hospitalization period. The time-course of Δglycosylation derived traits **(a)** fucosylation, **(b)** galactosylation, **(c)** bisection and **(d)** sialylation, as shown during hospitalization (n=111). Line colours correspond to a single COVID-19 patient, whilst the colour gradient in the circles/squares indicates the corresponding severity score (grey = NA). The shape displays whether a patient passed away (square) or was discharged alive (circle). The black dashed line with a grey 95% confidence interval band is a cubic polynomial fit over the shown datapoints to illustrate overall dynamics.

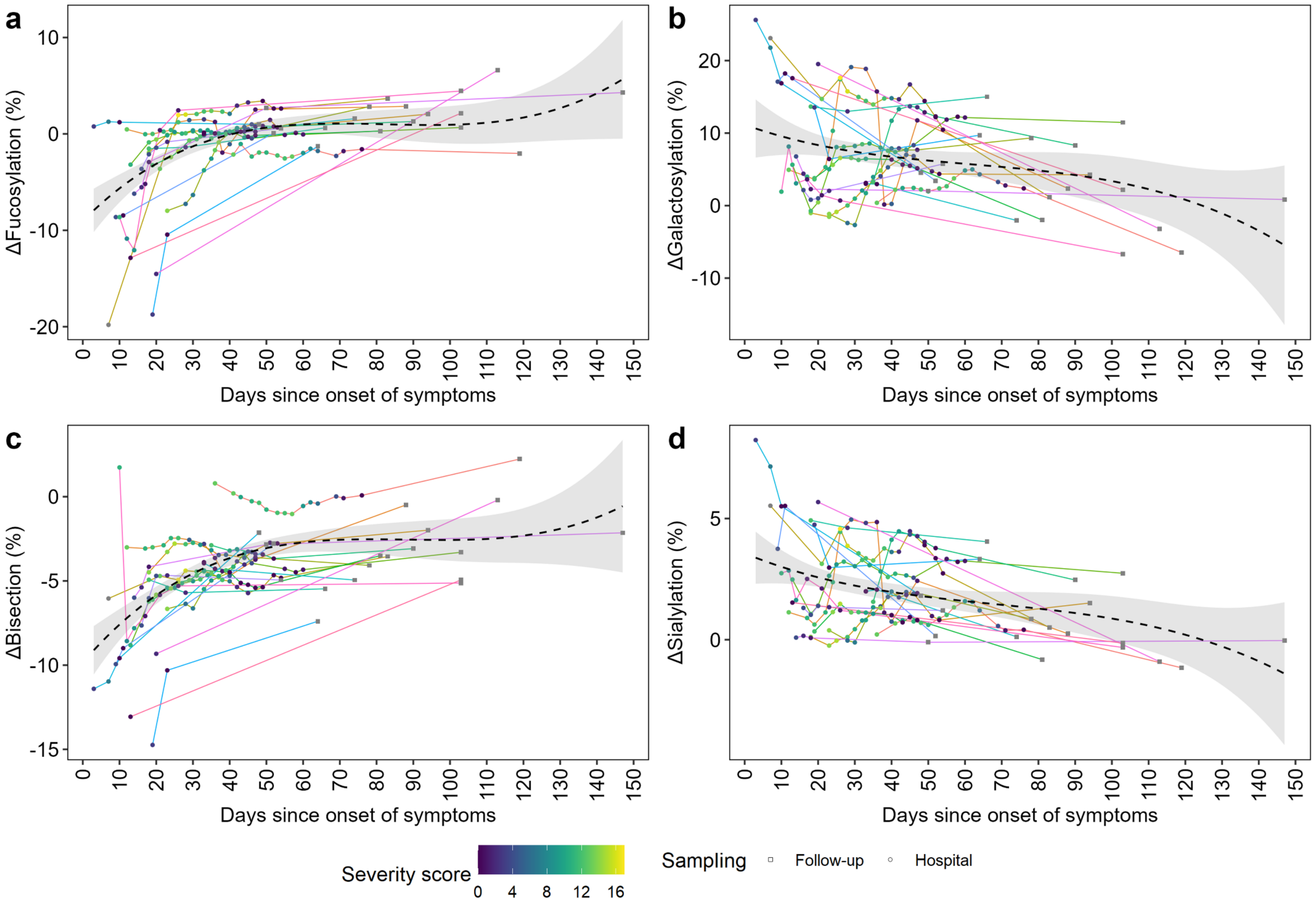
a
ΔFucosylation (%)
Days since onset of symptoms
b
ΔGalactosylation (%)
Days since onset of symptoms
c
ΔBisection (%)
Days since onset of symptoms
d
ΔSialylation (%)
Days since onset of symptoms
Severity score
0 4 8 12 16
Sampling
Follow-up
Hospital

o

Supplementary Fig. 5. ΔIgG1 glycosylation dynamics of the subset of patients with a follow-up sample. The time-course of Δglycosylation derived traits **(a)** fucosylation, **(b)** galactosylation, **(c)** bisection and **(d)** sialylation, as shown during the hospitalization period and follow-up (n=19). Line colours correspond to a single COVID-19 patient, whilst the colour gradient in the circles/squares indicates the corresponding severity score (grey = NA). The circle and shape display whether the timepoint corresponds to a follow-up sample (square) or to a sample taken during hospitalization (circle). The black dashed line with a grey 95% confidence interval band is a cubic polynomial fit over the shown datapoints to illustrate overall dynamics.

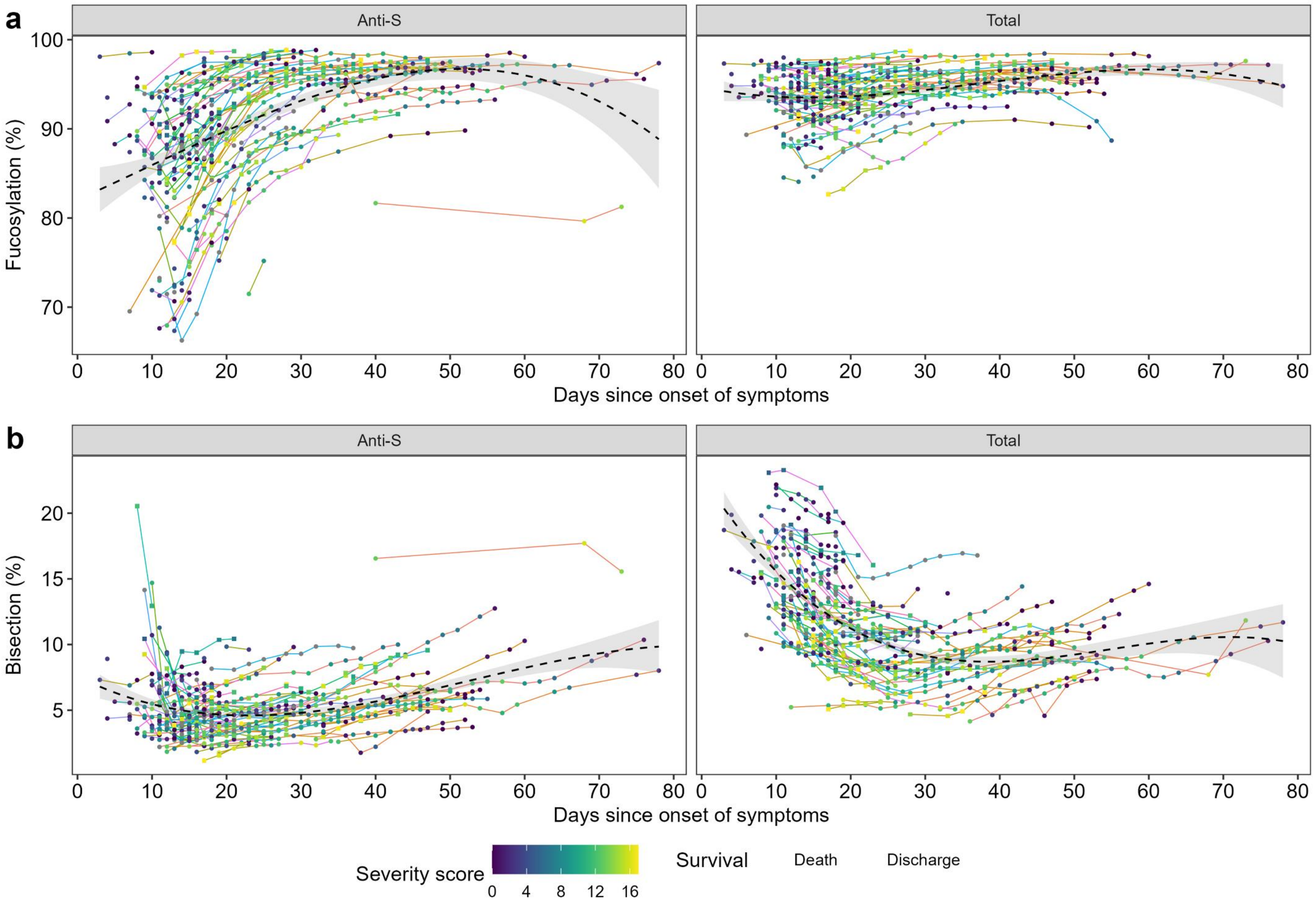
a
Anti-S
Total
Fucosylation (%)
Days since onset of symptoms
b
Anti-S
Total
Bisection (%)
Days since onset of symptoms
Severity score
0 4 8 12 16
Survival
Death
Discharge

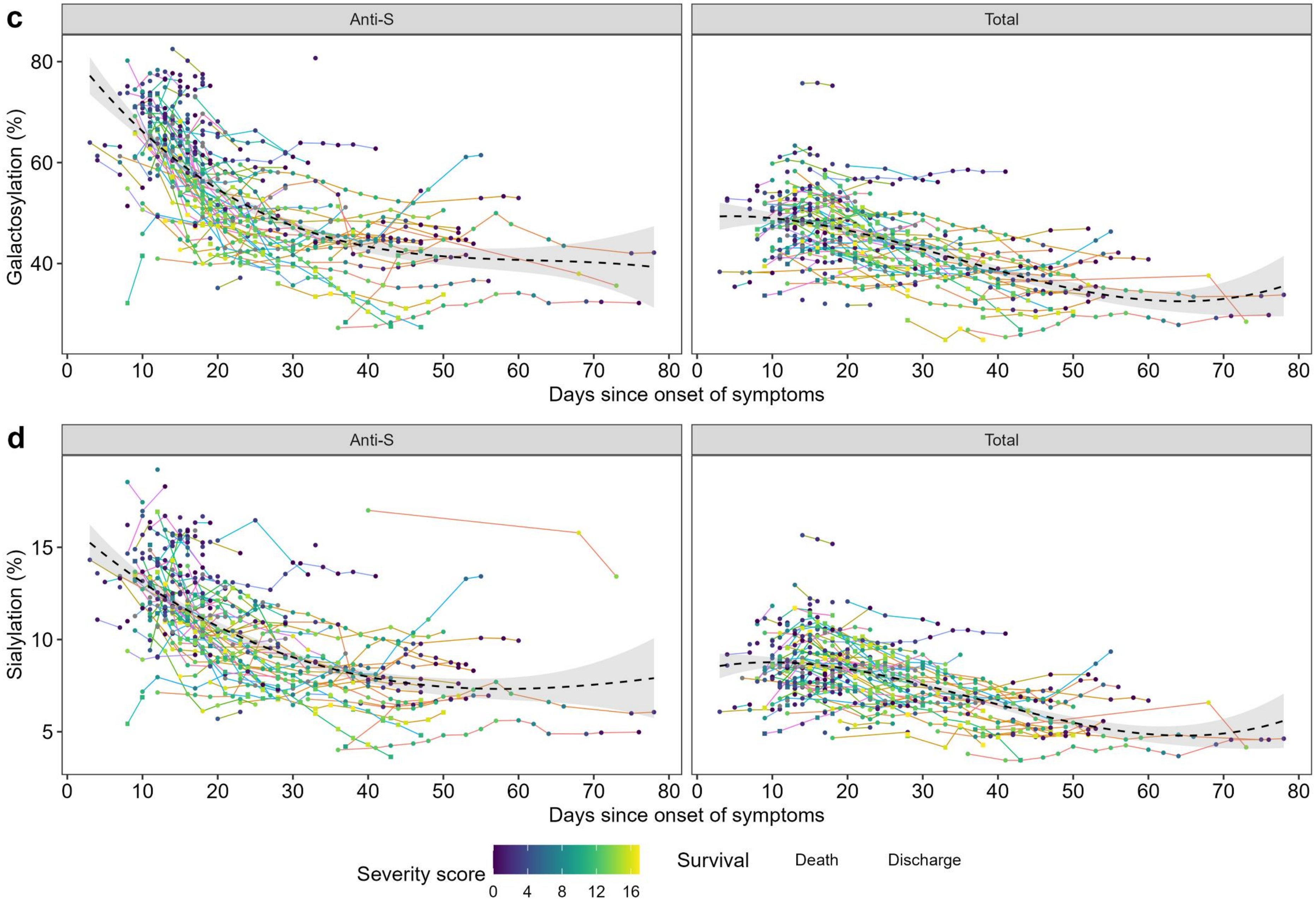
c
Anti-S
Total
Galactosylation (%)
80
60
40
0 10 20 30 40 50 60 70 80
Days since onset of symptoms
d
Anti-S
Total
Sialylation (%)
15
10
5
0 10 20 30 40 50 60 70 80
Days since onset of symptoms
Severity score
0 4 8 12 16
Survival
Death
Discharge

Supplementary Fig. 6. Anti-S and total IgG1 glycosylation dynamics during the entire hospitalization period. The time-course of glycosylation derived traits **(a)** fucosylation, **(b)** bisection, **(c)** galactosylation and **(d)** sialylation as shown during the hospitalization period (n=111). Anti-S IgG1 dynamics are shown in the left facets, whereas total IgG1 dynamics in the right facets in each panel. Line colors correspond to a single COVID-19 patient, whilst the colour gradient in the circles/squares indicates the corresponding severity score (grey = NA). The circle and shape display whether the patient passed away (square) or was discharged alive (circle) from the hospital. The black dashed line with a grey 95% confidence interval band is a cubic polynomial fit over the shown datapoints to illustrate overall dynamics. Note that the confounding effect of age largely influences the observed bisection **(Fig. S2c)**, galactosylation **(Fig. S2e)** and **(Fig. S2g)** sialylation pattern, thereby has been corrected for age effects **(Fig. S2)**.

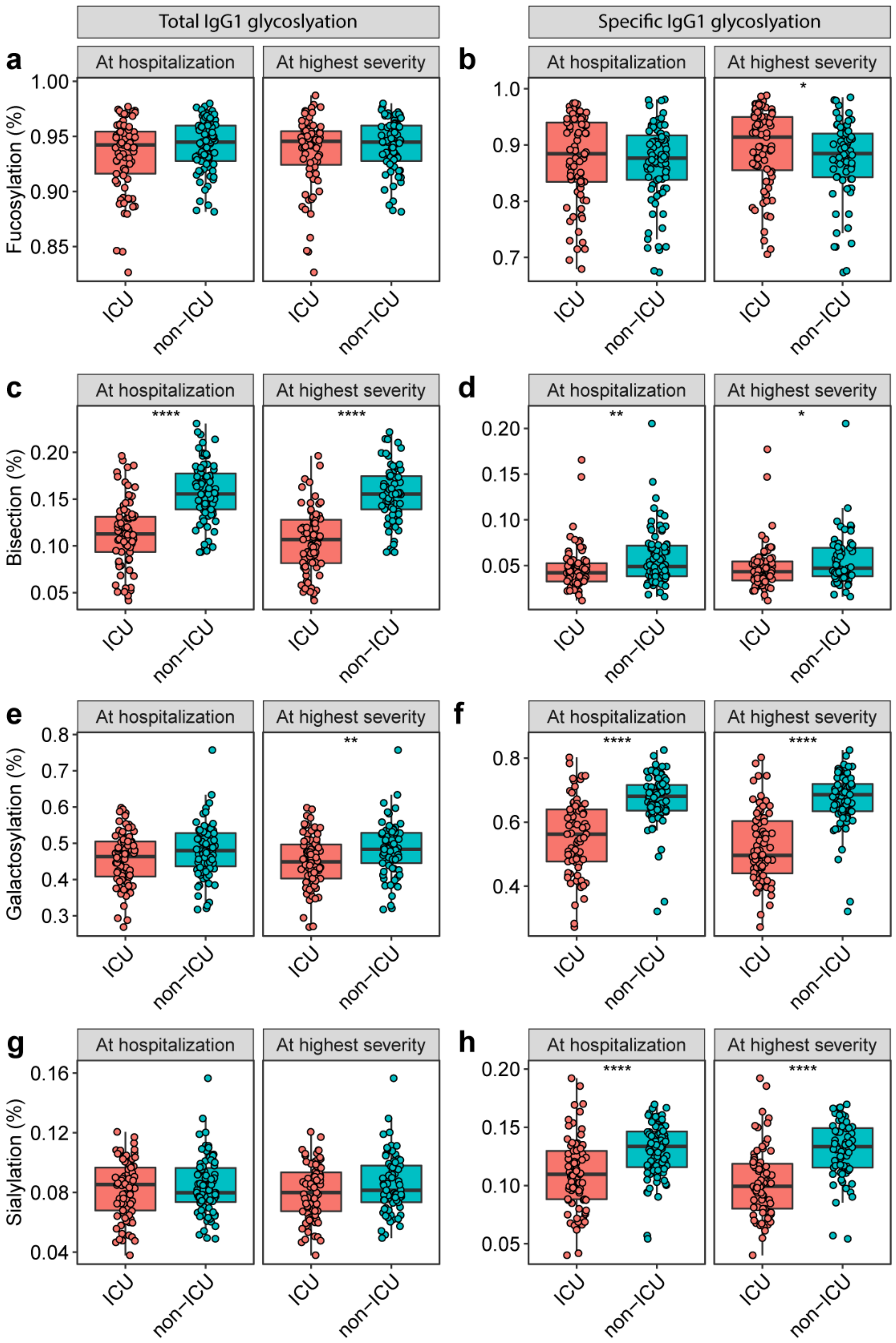
Total IgG1 glycoslyation
Specific IgG1 glycoslyation
a
b
c
d
e
f
g
h
At hospitalization
At highest severity
Fucosylation (%)
Bisection (%)
Galactosylation (%)
Sialylation (%)
ICU
non−ICU
*
**
****

Supplementary Fig. 7. Comparison of total (a, c, e, g) and anti-S (b, d, f, h) IgG1 glycosylation traits as per ICU admission.

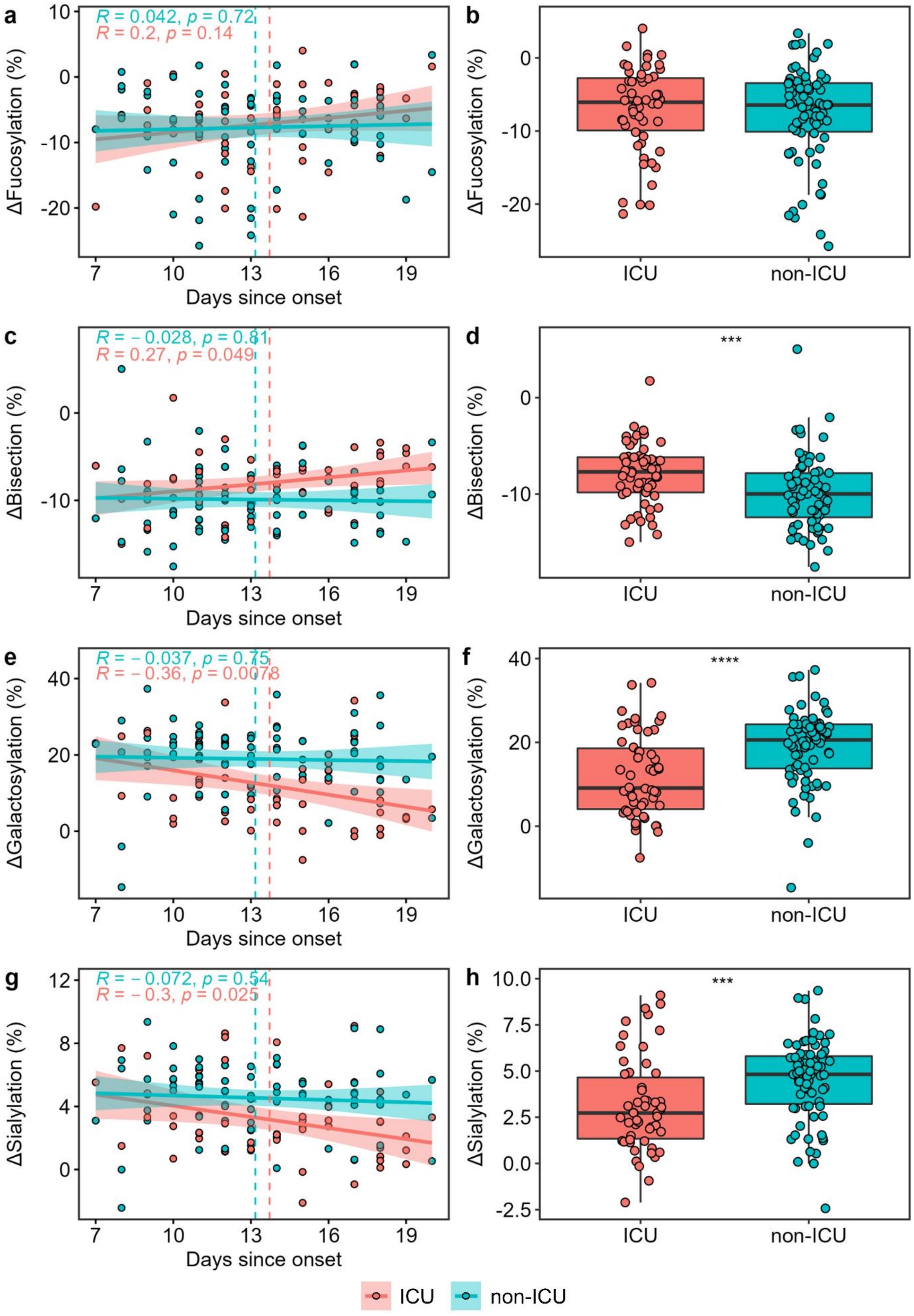

Supplementary Fig. 8. ICU (red) and non-ICU (blue) patients and corresponding ΔIgG1 glycosylation derived traits in a "days since onset of symptoms" subset of patients (n=129) to confirm that the observed differences (Fig. 3) are not confounded by vast glycosylation

dynamics. ΔIgG1 **(a)** fucosylation, **(c)** bisection, **(e)** galactosylation and **(g)** sialylation as a proxy of days since symptom onset. Shown in the inset are the Spearmen correlation coefficients (R) and *p*-values, respectively. The red (ICU) and blue (non-ICU) lines are linear regression lines and the corresponding band indicates the 95% confidence interval. The dashed vertical lines indicate group means. Comparison of corresponding ΔIgG1 **(b)** fucosylation, **(d)** bisection, **(f)** galactosylation and **(h)** sialylation levels between ICU and non-ICU patients. All datapoints correspond to baseline samples (time of hospitalization).

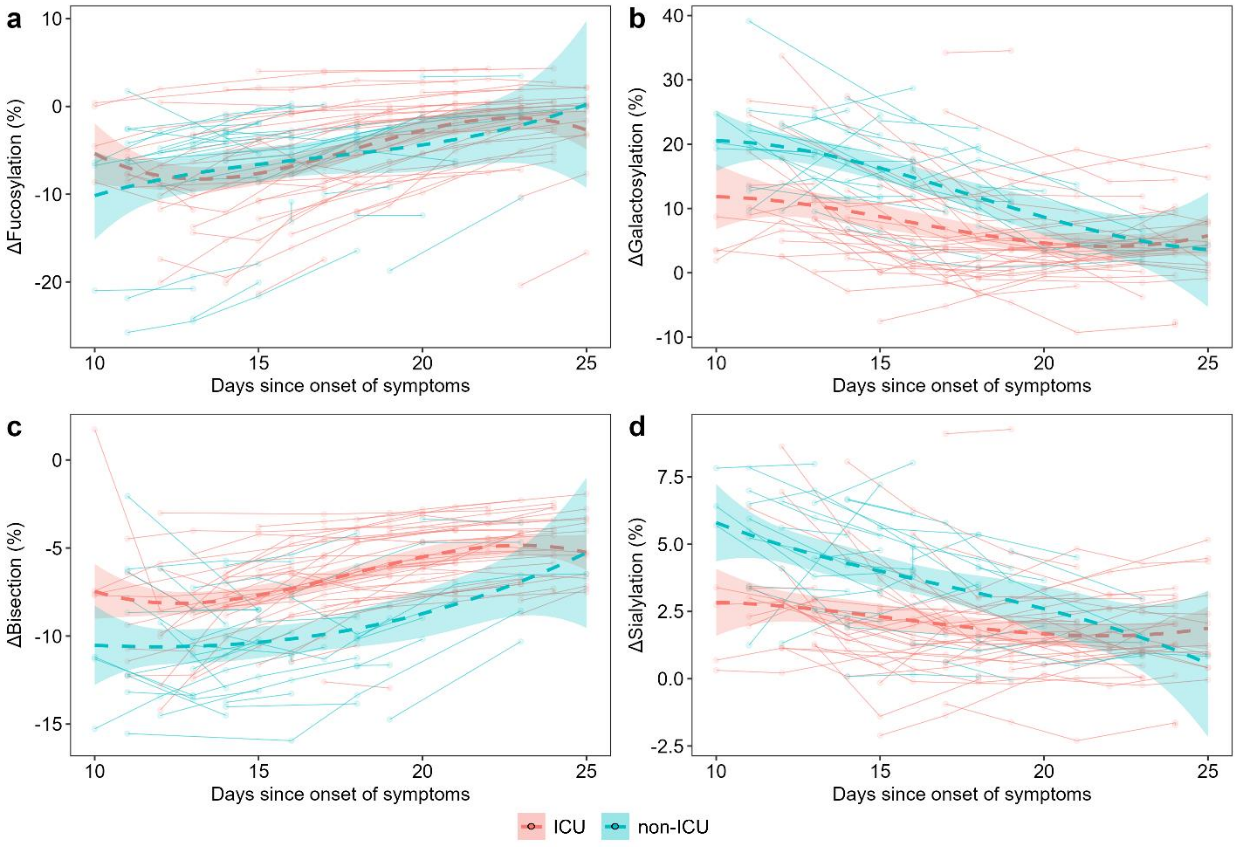

Supplementary Fig. 9. ΔIgG1 glycosylation dynamics of patients admitted to the ICU (n=48; red) and non-ICU (n=34; blue) treatments as shown between days 10 and 25. The time course of ΔIgG1 glycosylation traits **(a)** fucosylation, **(b)** galactosylation, **(c)** bisection and **(d)** sialylation. The dashed lines with 95% confidence interval bands are cubic polynomials fit over the shown datapoints to illustrate overall dynamics.

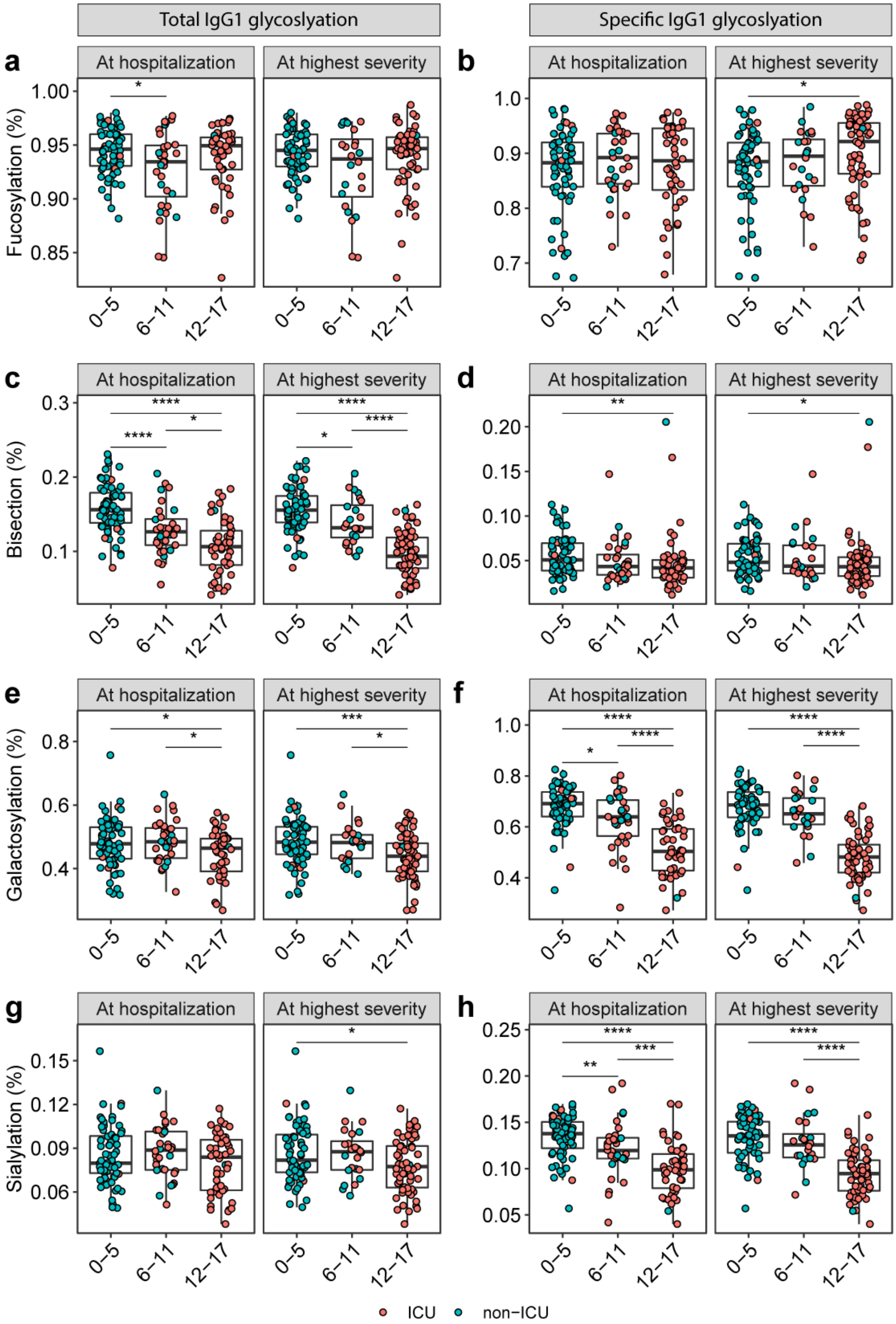

Supplementary Fig. 10. Comparison of total (a, c, e, g) and anti-S (b, d, f, h) IgG1 glycosylation traits as per severity score group.

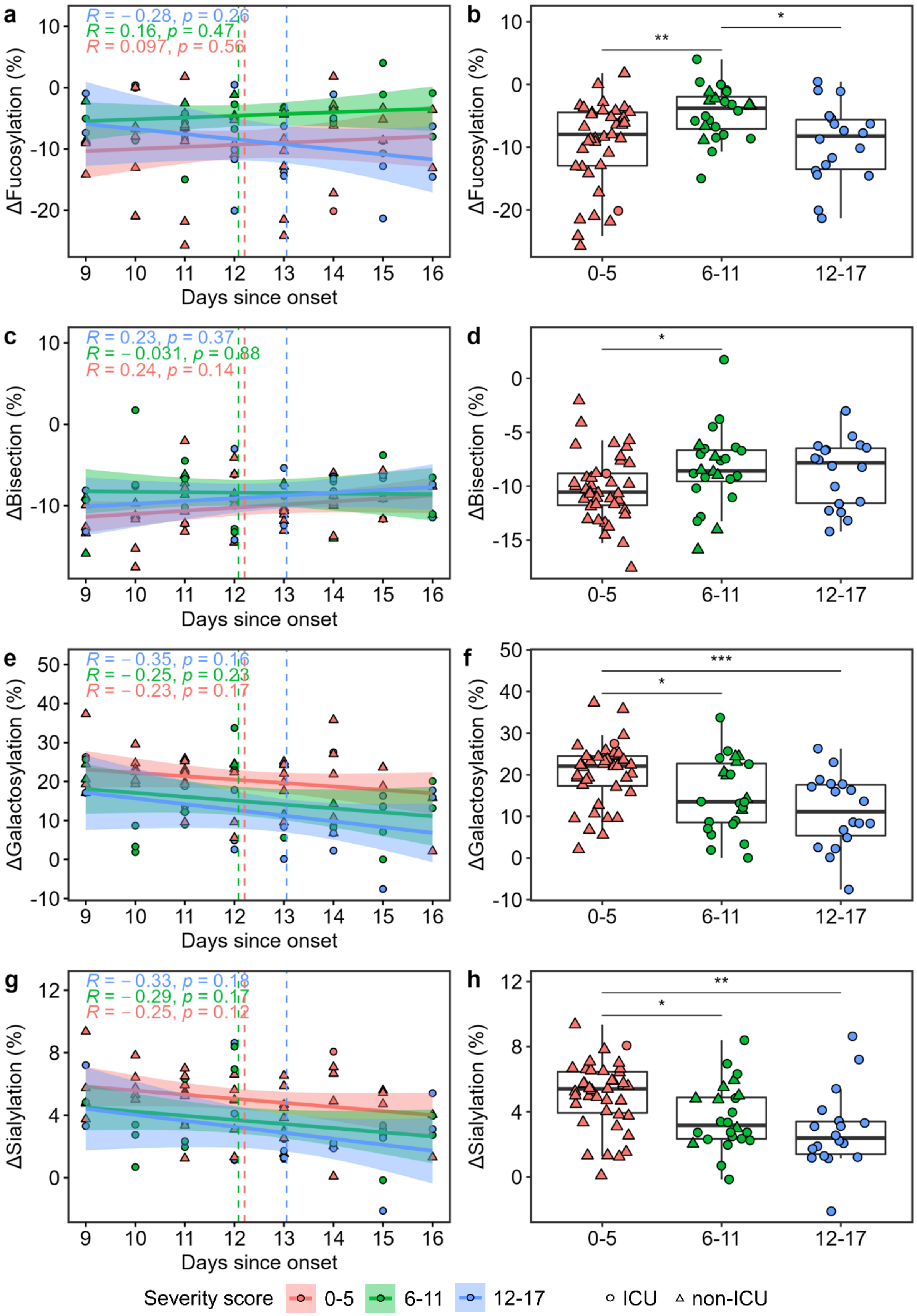
a
R = − 0.28, p = 0.26
R = 0.16, p = 0.47
R = 0.097, p = 0.56
ΔFucosylation (%)
Days since onset
b
ΔFucosylation (%)
0-5
6-11
12-17
c
R = 0.23, p = 0.37
R = − 0.031, p = 0.88
R = 0.24, p = 0.14
ΔBisection (%)
Days since onset
d
ΔBisection (%)
e
R = − 0.35, p = 0.16
R = − 0.25, p = 0.23
R = − 0.23, p = 0.17
ΔGalactosylation (%)
Days since onset
f
ΔGalactosylation (%)
g
R = − 0.33, p = 0.18
R = − 0.29, p = 0.17
R = − 0.25, p = 0.12
ΔSialylation (%)
Days since onset
h
ΔSialylation (%)
Severity score
0-5
6-11
12-17
ICU
non-ICU

Supplementary Fig. 11. Patients in varying severity score groups 0-5 (red), 6-11 (green) and 12-17 (dark blue) and corresponding ΔIgG glycosylation derived traits in a "days since onset of symptoms" subset of patients to confirm that the observed differences (Fig. 4) are not confounded by vast glycosylation dynamics. ΔIgG1 **(a)** fucosylation and **(c)** bisection, **(e)** galactosylation and **(g)** sialylation as a proxy of days since onset of subset of patients. Shown in the inset are the Spearmen correlation coefficients (R) and *p*-values, respectively. The red (0-5), green (6-11) and blue (12-17) lines are linear regression lines and the corresponding band indicates the 95% confidence interval. Dashed vertical lines indicate group mean. Comparison of corresponding ΔIgG1 **(b)** fucosylation and **(d)** bisection, **(f)** galactosylation and **(h)** sialylation levels between the three severity score groups. Circle indicates ICU patients, whereas squares indicate non-ICU patients. All datapoints correspond to baseline samples (time of hospitalization).

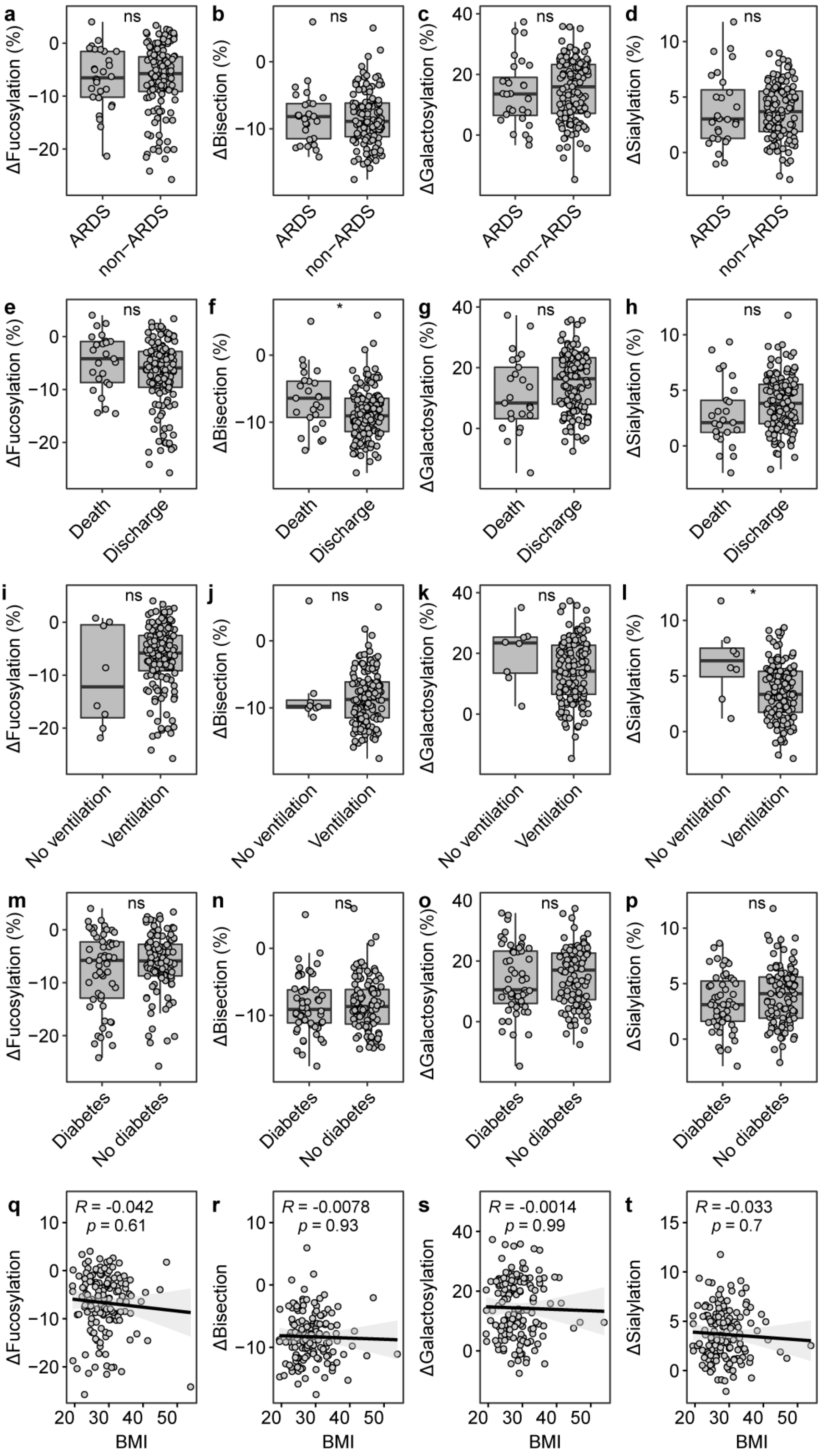
a
ΔFucosylation (%)
ns
ARDS
non-ARDS
b
ΔBisection (%)
ns
c
ΔGalactosylation (%)
ns
d
ΔSialylation (%)
ns
e
ns
Death
Discharge
f
*
g
ns
h
ns
i
ns
No ventilation
Ventilation
j
ns
k
ns
l
*
m
ns
Diabetes
No diabetes
n
ns
o
ns
p
ns
q
R = -0.042
p = 0.61
ΔFucosylation
BMI
r
R = -0.0078
p = 0.93
ΔBisection
s
R = -0.0014
p = 0.99
ΔGalactosylation
t
R = -0.033
p = 0.7
ΔSialylation

Supplementary Fig. 12. Comparison of acute respiratory distress syndrome (a-d), survival (e-h) and ventilation (i-l), diabetes (m-p) subgroups and BMI (q-t) of patients for Δglycosylation traits fucosylation (a, e, i, m, q), bisection (b, f, j, n, r), galactosylation (c, g, k, o, s) and sialylation (d, h, l, p, t). Bisection negatively associated with, survival, and sialylation negatively associated with ventilation. No other associations were found.

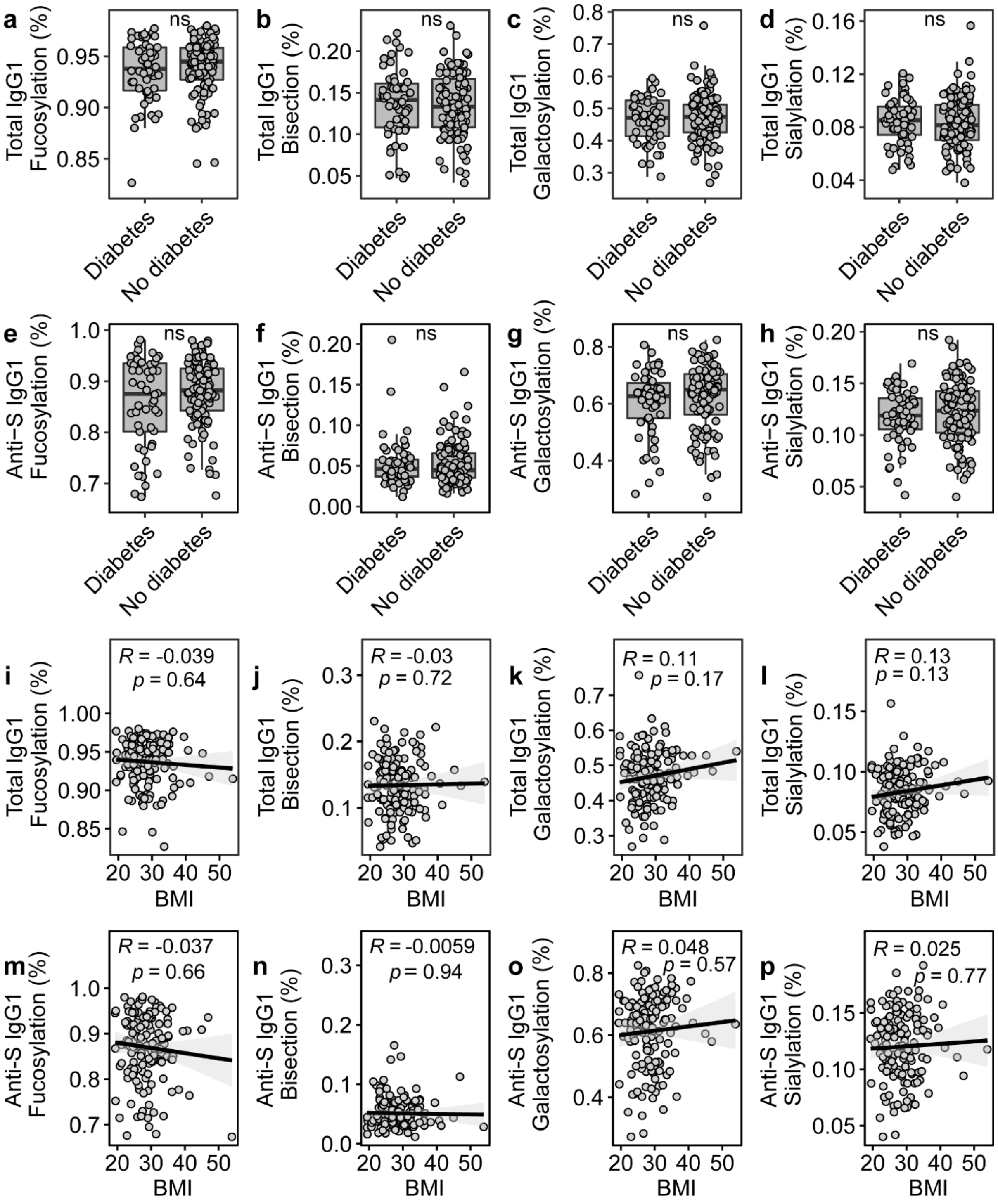

Supplementary Fig. 13. Comparison of diabetes (a-h) subgroups and BMI (i-o) of patients for total and anti-S IgG1 glycosylation traits fucosylation (a, e, i, m), bisection (b, f, j, n),

galactosylation (c, g, k, o) and sialylation (d, h, l, p). In contrast to our findings, total IgG bisection, galactosylation and sialylation have been described to associate with diabetes[2] and BMI[3]. Of note, diabetes and obesity are chronic conditions, which can hardly be compared to the current acute infection setting characterized by massive pathogen-specific antibody neo-production with a transient, high galactosylation (and sialylation) phenotype against a broad range of antigens, as described elsewhere[4]. Moreover, the same studies did not differentiate between Fc and Fab glycosylation or IgG subclasses[2,3]. Furthermore, the current study mainly involves an older patient group **(Table 1)**, whereas the referred previous studies comprised a very large number of participants with a broad age range, and involved data processing steps and methods to account for confounders that were inherently different from the current approach[2,3]. These factors may explain the observed differences and may hamper a conclusive comparison to previous literature on diabetes and BMI and their associations with IgG glycosylation. Anti-S IgG1 glycosylation traits likewise showed no associations with BMI and diabetes.

References

1. Gudelj I, Lauc G, Pezer M. Immunoglobulin G glycosylation in aging and diseases. *Cell Immunol* 2018; **333**: 65-79.
2. Lemmers RFH, Vilaj M, Urda D, et al. IgG glycan patterns are associated with type 2 diabetes in independent European populations. *Biochim Biophys Acta Gen Subj* 2017; **1861**(9): 2240-9.
3. Nikolac Perkovic M, Pucic Bakovic M, Kristic J, et al. The association between galactosylation of immunoglobulin G and body mass index. *Prog Neuropsychopharmacol Biol Psychiatry* 2014; **48**: 20-5.
4. Selman MH, de Jong SE, Soonawala D, et al. Changes in antigen-specific IgG1 Fc N-glycosylation upon influenza and tetanus vaccination. *Mol Cell Proteomics* 2012; **11**(4): M111 014563.
